# The impact of genome evolution on the allotetraploid *Nicotiana rustica* – an intriguing story of enhanced alkaloid production

**DOI:** 10.1186/s12864-018-5241-5

**Published:** 2018-11-29

**Authors:** N. Sierro, J. N. D. Battey, L. Bovet, V. Liedschulte, S. Ouadi, J. Thomas, H. Broye, H. Laparra, A. Vuarnoz, G. Lang, S. Goepfert, M. C. Peitsch, N. V. Ivanov

**Affiliations:** Philip Morris International R&D, Philip Morris Products S.A, 2000 Neuchatel, Switzerland

**Keywords:** Nicotiana, Polyploidy, Speciation, Genome, Evolution, Comparative genomics

## Abstract

**Background:**

*Nicotiana rustica* (Aztec tobacco), like common tobacco (*Nicotiana tabacum*), is an allotetraploid formed through a recent hybridization event; however, it originated from completely different progenitor species. Here, we report the comparative genome analysis of wild type *N. rustica* (5 Gb; 2n = 4x = 48) with its three putative diploid progenitors (2.3–3 Gb; 2n = 2x =24), *Nicotiana undulata*, *Nicotiana paniculata* and *Nicotiana knightiana*.

**Results:**

In total, 41% of *N. rustica* genome originated from the paternal donor (*N. undulata*), while 59% originated from the maternal donor (*N. paniculata*/*N. knightiana*). Chloroplast genome and gene analyses indicated that *N. knightiana* is more closely related to *N. rustica* than *N. paniculata*. Gene clustering revealed 14,623 ortholog groups common to other Nicotiana species and 207 unique to *N. rustica*. Genome sequence analysis indicated that *N. knightiana* is more closely related to *N. rustica* than *N. paniculata*, and that the higher nicotine content of *N. rustica* leaves is the result of the progenitor genomes combination and of a more active transport of nicotine to the shoot.

**Conclusions:**

The availability of four new Nicotiana genome sequences provide insights into how speciation impacts plant metabolism, and in particular alkaloid transport and accumulation, and will contribute to better understanding the evolution of Nicotiana species.

**Electronic supplementary material:**

The online version of this article (10.1186/s12864-018-5241-5) contains supplementary material, which is available to authorized users.

## Background

While *Nicotiana tabacum* is the most notable species from the Nicotiana genus, various other Nicotiana species are cultivated as crops, grown as ornamental garden plants or used as model organisms in research. Aztec or Indian tobacco, *Nicotiana rustica*, is suspected to be the original tobacco species that was brought from the Americas to Europe. Known as “*mapacho*”, it was considered sacred and medicinal by Amazonian shamans. Even though, in terms of production, it has been superseded in the last century by its relative *N. tabacum*, Aztec tobacco is still cultivated in South America, Turkey, Russia and Vietnam, mostly owing to its resilience to adverse climatic conditions.

Morphologically, *N. rustica* is recognized for its characteristic yellow flowers that form a tube (Additional file 1: Figure S1) and leaves that are covered with trichomes rich in secondary metabolites, including nicotine, nornicotine, anatabine and anabasine [1]. The high leaf concentration of nicotine (5–15% dry leaf weight) prompted its use in the production of nicotine-based pesticides, nicotine sulfate and nicotinic acid. Because of even higher levels of citric acid (15–20% of dry leaf weight), the leaves of *N. rustica* are an excellent source of this important metabolite [2]. Scientific reports describing active accumulation of nicotine in *N. rustica* compared to *N. tabacum* are rather scarce [3, 4]. Interestingly, on the opposite to the nicotine level *N. rustica* exhibits a lower leaf versus root cadmium ratio compared to *N. tabacum* [5, 6]. As root is both involved in nicotine synthesis, cadmium uptake and shoot translocation, root pathways may have interconnection, Cd being reported to have toxic properties regarding plant nutrition [7, 8]. On the side of Cd, although Zn accumulation, but not Fe and Mn, may vary between the two species [6], no reports mention yet variation of K and Na.

Within the Solanaceae family, the genomes of the Nicotiana species are peculiar. First, they have relatively large genomes that are similar in size to those of Capsicum species and two to three times larger than those of Solanum and Petunia species. Second, the Nicotiana genus contains many species that can be used to study the evolution of polyploidy in plants. Although the majority of the more than 70 Nicotiana species is diploid with *n* = 12, five sections of the Nicotiana family (*Nicotiana*, *Polydicliae*, *Repandae*, *Undulatae* and *Rusticae*) include allopolyploid species with *n* = 24 [9]. Molecular clock analyses estimate the dates of polyploidization events as ranging from less than 0.2 million years ago (*Nicotiana arentsii*, *N. rustica* and *N. tabacum*) to more than 10 million years ago (a single polyploidization event from which sect. *Suaveolentes* is descended) [9–11]. To date, only the progenitor species of *N. tabacum* (*Nicotiana sylvestris* and *Nicotiana tomentosiformis*) have been well characterized [12], and the presence of previously identified species-specific translocations in *N. tabacum* [13, 14] have been confirmed [15].

Based on morphology, cytology and artificial hybridization experiments, Goodspeed [16] proposed the likely progenitors of the polyploid species of the Nicotiana genus. The origins of 15 allopolyploid *Nicotiana* species were explored by genomic in situ hybridization (GISH) [17], and fluorescently labeled DNA probes from the genomes of *N. undulata* and *N. paniculata* marked the complimentary chromosomes of *N. rustica,* confirming Goodspeed's hypothesis of their parental relationships. Lim et al. [18] investigated genome evolution in three natural allopolyploid species (*N. arentsii*, *N. rustica* and *N. tabacum*) using GISH and fluorescent in situ hybridization. Unlike in *N. tabacum* cultivars, no intergenomic translocations were observed in *N. rustica*; thus, the probes from *N. undulata* and *N. paniculata* have been exclusively mapped to the U- and P-genomes, respectively, of *N. rustica*.

Using complementary PCR-based techniques and the internal transcribed spacer sequences of nuclear ribosomal DNA [17] and the chloroplast gene *matK* [19], the parental relationships of species were assessed. They provided further evidence that either *N. knightiana* or *N. paniculata* could be the maternal donor, and both techniques identified *N. undulata* as the paternal donor. Interestingly, *N. undulata* serves as a maternal donor in the hybridization with *Nicotiana wigandioides*, to form the allotetraploid *N. arentsii* [17, 19].

Unfortunately, *N. knightiana* was not investigated using GISH. Based on a screen of 75 Nicotiana species with several chloroplast genes, Clarkson et al. [10] established that *N. knightiana* is genetically closer than *N. paniculata* (one vs five substitutions) to *N. rustica*. Nevertheless, this still suggested that a common ancestor of both *N. knightiana* and *N. paniculata* served as the maternal donor to *N. rustica*. Thus, to date, the identity of the progenitor species from section Paniculatae that is the maternal donor to the *N. rustica* genome remains unclear. Our analysis of the chloroplast genomes from all four species shed light on this topic.

The genomes of *Nicotiana benthamiana* [20, 21], *N. otophora* [15], *N. sylvestris* [12], *N. tabacum* [15, 22], *N. tomentosiformis* [12], *N. attenuata* [23] and *N. obtusifolia* [23] have been sequenced and draft assemblies published, enabling genome-based evolutionary studies of Nicotiana species. With the exception of *N. benthamiana*, *N. attenuata* and *N. obtusifolia*, all of the published Nicotiana genomes are closely related to *N. tabacum*. Here, we present the genomes and transcriptomes of *N. rustica* and its putative ancestral species, *N. undulata*, *N. paniculata* and *N. knightiana*. We elucidate the mechanism behind the upregulated nicotine production in these plants and provide insights into the metabolic and genomic differences in comparison with *N. tabacum* and its ancestors, *N. sylvestris* and *N. tomentosiformis*, also focusing on the accumulation of essential and non-essential elements as well as major free amino acids, no studies being available yet.

## Results

### Genome sequencing, assembly and annotation

We sequenced the genomes of *N. rustica* and its potential progenitors, *N. undulata*, *N. paniculata* and *N. knightiana*, using reads from Illumina HiSeq2500 and Pacific Biosciences RSII sequencers and performed de novo genome assemblies. The estimations of their genome sizes based on 31-k-mer depth distributions of raw sequencing reads were 4.99 Gb for *N. rustica*, 2.18 Gb for *N. undulata*, 3.26 Gb for *N. paniculata* and 3.12 Gb for *N. knightiana*, which are consistent with the sizes (5.181, 2.362, 2.880 and 3.090 Gb, respectively) reported in the KEW c-DNA database. Based on these numbers, the genome of *N. rustica* was reduced by 5.9 or 8.3% compared with the sum of the genome sizes from *N. undulata* and either *N. paniculata* or *N. knightiana*, respectively. This reduction corresponds to the upper bound proposed by Leitch et al. [9] and is similar to the reduction in genome size reported for *N. tabacum* [15].

The assembled genome sequences consisted of 117,559 to 246,567 scaffolds, covering from 67.2 to 89.2%, respectively, of the estimated genomes. The N50 lengths were between 52.8 and 84.6 kb (Table 1). The comparatively low k-mer-based genome coverage of *N. paniculata* (67.2%) results from the overestimation of its genome size when using 31-k-mers. While the estimation methods produced similar genome sizes for the other species, this was not the case for *N. paniculata*, for which flow cytometric measurements resulted in a smaller estimated genome size.

**Table 1:** Nicotiana genome assembly metrics

	*N. rustica*	*N. undulata*	*N. paniculata*	*N. knightiana*
Coverage	90x	122x	100x	82x
Contigs	863,445	240,808	289,247	368,273
Scaffolds	246,567	117,559	181,973	160,417
Average scaffold length	18,053	16,284.2	12,038.2	14,331.7
Longest scaffold	709,624	435,587	551,851	749,769
N50 length	84,603	61,881	52,808	82,722
E-size	106,321.7	76,855.5	68,024.3	103,550.5
Assembly length	4,451,279,893	1,914,350,984	2,190,627,570	2,299,051,887
Undefined bases	458,861,024 (10.31%)	15,815,547 (0.83%)	2,994,201 (0.14%)	47,367,942 (2.06%)
Genome size (KMER) [Gb]	4.99	2.18	3.26	3.12
Genome size (KEW) [Gb]	5.181	2.362	2.88	3.09
Genome coverage (KMER)	0.892	0.878	0.672	0.737
Genome coverage (KEW)	0.8591	0.81	0.761	0.744
genes and regulatory sequences	956,876,918 (24%)	469,801,265 (25%)	487,609,360 (22%)	513,178,366 (23%)
DNA transposons	107,092,333 (3%)	56,581,894 (3%)	55,826,622 (3%)	55,840,784 (2%)
LTR elements	1,747,332,959 (44%)	820,008,945 (43%)	981,515,044 (45%)	1,014,595,360 (45%)
Retrotransposons	532,753,114 (13%)	243,526,350 (13%)	303,566,434 (14%)	304,961,922 (14%)
Non-LTR retroelements (SINES, LINES)	30,145,844 (1%)	15,712,962 (1%)	15,526,693 (1%)	15,258,713 (1%)
Others (Satellites, unknown, low complexity)	618,217,701 (15%)	292,904,021 (15%)	343,589,216 (16%)	347,848,800 (15%)

### Genome repeat contents

An analysis of the assembled genomes organization showed that 22 to 25% of the sequenced genomes consisted of genes and regulatory sequences (Table 1). The 75 to 78% of the genomes identified as repeats consisted, to a large extent, of long terminal repeat elements (~ 45% of the genome assemblies). The proportions of each type of repeat element found in *N. rustica*, *N. undulata*, *N. knightiana* and *N. paniculata* were very similar. They also correspond to those previously observed in *N. tabacum* and its ancestors [12, 15].

Lim et al. [18, 24] used GISH and fluorescent in situ hybridization to detect rearrangements and at least 10-fold reductions in the NPAMBO repeat content within the P-genome of *N. rustica* compared within that of *N. paniculata*. Based on the draft genome assemblies, no such reduction in the NPAMBO repeat element was observed (Additional file 2: Table S1).

### Genome assembly completeness

The completeness of the genomes was assessed using Benchmarking Universal Single-Copy Orthologs (BUSCO) with the embryophyta plant dataset, which consists of 1440 universal single-copy orthologs [25]. Additional file 3: Figure S2 shows the percentage of these universal single-copy orthologs that were identified as complete, duplicated, fragmented and missing in Nicotiana genomes, other Solanacea genomes, *Vitis vinifera* and *Arabidopsis thaliana*.

Most diploid Nicotiana species contain approximately 95% complete universal single-copy orthologs (approximately 90% as single copies and 5% as duplicates), similar to other diploid Solanacea species and *A. thaliana*. *N. otophora* is the only exception, with approximately 75% single copied and 5% duplicated complete universal single-copy orthologs. It also contains approximately 10% fragmented universal single-copy orthologs, which is higher than in the other genomes.

The tetraploid Nicotiana species also contain approximately 95% complete universal single-copy orthologs. However, the proportions of single copies and duplicates are very different, with only approximately 30% of single copies complete universal single-copy orthologs being contained in the recent tetraploids *N. tabacum* and *N. rustica*, and approximately 50% in the more ancient tetraploid *N. benthamiana*.

### Parental origins based on raw sequencing data

**Fig. 1:**
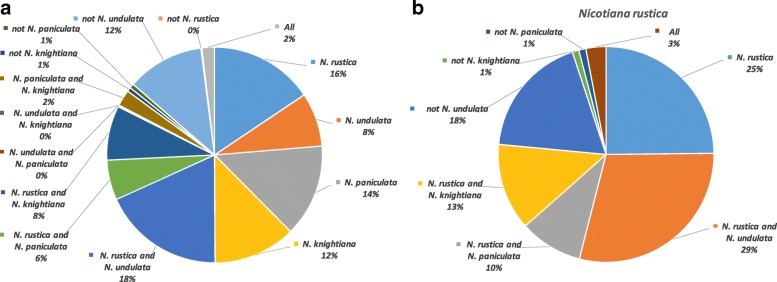
Parental origins of *N. rustica* based on the 31-k-mer analysis. **a** 31-k-mers from *N. rustica* and its progenitors; **b** 31-k-mers from *N. rustica* only

The 31-k-mers present in the raw sequencing reads of *N. rustica*, *N. undulata*, *N. paniculata* and *N. knightiana* between 5 and 250 times were compared to evaluate the overlap among the four species (Fig. 1a). Approximately 50% of them are unique to one species and only 2% are common to all four species; 18% are common to *N. rustica* and *N. undulata*, and 26% to *N. rustica* and *N. paniculata* and/or *N. knightiana* (12% with both species, 8% with *N. knightiana* and 6% with *N. paniculata*).

Of the 31-k-mers found in the *N. rustica* raw reads (Fig. 1b), 25% are unique to *N. rustica*, 29% are shared with *N. undulata*, and 41% with *N. paniculata* and/or *N. knightiana*.

The proportion of 31-k-mers found in one of the two ancestors indicates that 41.4% of the *N. rustica* genome originated from *N. undulata* and 58.6% from *N. paniculata* and/or *N. knightiana*, which is consistent with the genome contributions of each ancestor to the hybridization (40.6 and 59.4%, respectively, calculated using predicted genome sizes of 2.18 Gb for *N. undulata*, 3.19 Gb for *N. paniculata*/*N. knightiana* (average of the predicted 3.12 Gb and 3.26 Gb genome sizes), and 5.37 Gb for *N. rustica* at the time of hybridization). Based on an estimated *N. rustica* genome of 4.99 Gb, the 41.4% contributed by *N. undulata* accounted for 2.07 Gb (0.11 Gb downsizing from the estimated *N. undulata* genome size), and *N. paniculata* and *N. knightiana* accounted for the remaining 2.92 Gb (0.27 Gb downsizing from the average of the estimated *N. paniculata* and *N. knightiana* genome sizes).

In comparison, in *N. tabacum*, the 31-k-mer analysis showed that 40.3% of the genome originated from *N. tomentosiformis* and 59.7% from *N. sylvestris*, whereas the estimated genome sizes of the ancestors indicated contributions to the hybridization of 46.2 and 53.8%, respectively, calculated with genome sizes of 2.22 Gb for *N. tomentosiformis* and 2.59 Gb for *N. sylvestris*, and 4.81 Gb for *N. tabacum* at the time of hybridization*.* Based on an estimated *N. tabacum* genome size of 4.41 Gb [15], the 40.3% contributed by *N. tomentosiformis* accounted for 1.78 Gb (0.44 Gb downsizing from the estimated *N. tomentosiformis* genome size), and *N. sylvestris* accounted for the remaining 2.63 Gb (0.04 Gb upsizing from the estimated *N. sylvestris* genome size) [12].

### Maternal parent of *N. rustica* based on a chloroplast genome analysis

The maternal parent of the tetraploid *N. rustica* was identified as being the ancestor of *N. paniculata* and *N. knightiana* by mapping the short sequencing reads from *N. rustica* and its ancestors to the chloroplast genome of *N. tabacum* [26] and by assessing the number of single nucleotide polymorphisms (SNPs) shared between *N. rustica* and *N. undulata*, and between *N. rustica* and *N. paniculata* and/or *N. knightiana*. Additional file 4: Figure S3 shows the overlap of the chloroplast genomes from the four Nicotiana species based on the number of common SNPs. We observed 336 SNPs unique to *N. undulata*, 8 shared between *N. rustica* and *N. undulata*, 303 SNPs shared by *N. rustica*, *N. paniculata* and *N. knightiana*, 7 by *N. rustica* and *N. paniculata*, 17 by *N. rustica* and *N. knightiana* and 11 by *N. paniculata* and *N. knightiana*. The chloroplast genome of *N. knightiana* appears to be closer than that of *N. paniculata* to the *N. rustica* chloroplast genome.

### Transcriptomics analysis

**Fig. 2:**
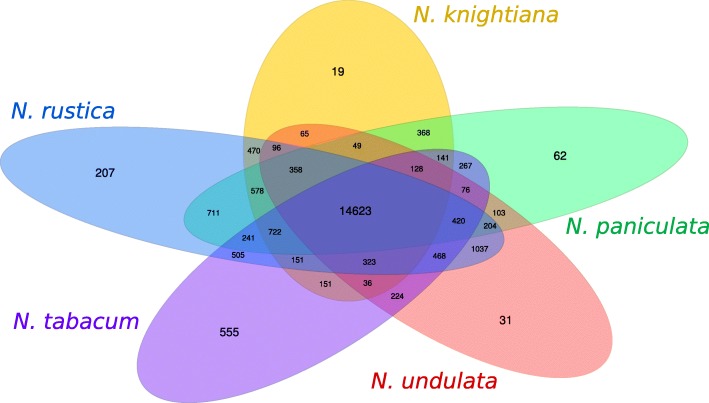
Numbers of OrthoMCL clusters of orthologous proteins based on RNA sequencing data

For each species, samples were taken from various organs under different conditions and at different time points. For *N. rustica*, two sets of transcriptomes were generated, one including eight tissues at a single time point, (flower bud, mature flower, mature capsule, lower leaf, middle leaf, upper leaf, stem and root) and the other one containing only root, upper and lower leaves and flower. For the putative progenitors, only the four-tissue set was generated. Expressed gene families were assigned using OrthoMCL. The gene families from the four-tissue set were used to ensure the comparability of datasets. As shown in Fig. 2, there is a common core of 14,623 ortholog groups that is shared by all species. As evidenced by the 207 ortholog groups specific to *N. rustica*, it has experienced only a minor divergence, as have the pseudo-progenitor species, each having fewer than 62 exclusive ortholog groups. *N. rustica* shares 1037 ortholog groups exclusively with *N. undulata*. Because *N. paniculata* and *N. knightiana* have diverged from the most recent common ancestor, the share of exclusive common orthologs is split between the two pseudo-progenitors, and *N. paniculata* appears closer to *N. rustica* (711) than *N. knightiana* (470 groups).

No potential regulators or genes of the nicotine biosynthesis pathway were identified in clusters of orthologous genes that are specific to either *N. tabacum* or *N. rustica* based on annotation assigned by blastp searches against TAIR and ITAG proteins, as well as against key regulators and genes of the nicotine biosynthesis pathway. Similarly, no potential regulators or genes of the nicotine biosynthesis pathway were identified in clusters of orthologous genes that are shared between *N. rustica* and *N. undulata*, or *N. rustica* and *N. paniculata* and/or *N. knightiana*, that could have provided insights on the maternal and paternal progenitors impact on the alkaloid pathway.

### Core gene set for phylogenetic analysis

To obtain a gene set for the phylogenetic analysis, OrthoMCL was run using the groups mentioned above, as well as the ancestors of tobacco, *N. sylvestris* and *N. tomentosiformis*, with tomato as an outgroup. A core set of 12,401 ortholog groups is shared among the species. Of these, 3041 have a group composition that corresponds to the expectations of polyploidization: one group member for the diploid species and two members for the allotetraploids. These can be aligned and used to calculate phylogenetic properties. A filtered subset of 2951 groups showed that 1250 *N. rustica* genes clustered with genes from *N. knightiana*, 1048 *N. rustica* genes clustered with genes from *N. paniculata*, and 653 *N. rustica* genes clustered with genes from the common ancestor of *N. knightiana* and *N. paniculata*. This result supports the earlier observation, based on the chloroplast genome SNP analysis, that *N. knightiana* is closer to *N. rustica* than *N. paniculata* and corroborates the phylogenic tree constructed by Sarkinen et al. [27].

### Alkaloid pathways

**Fig. 3:**
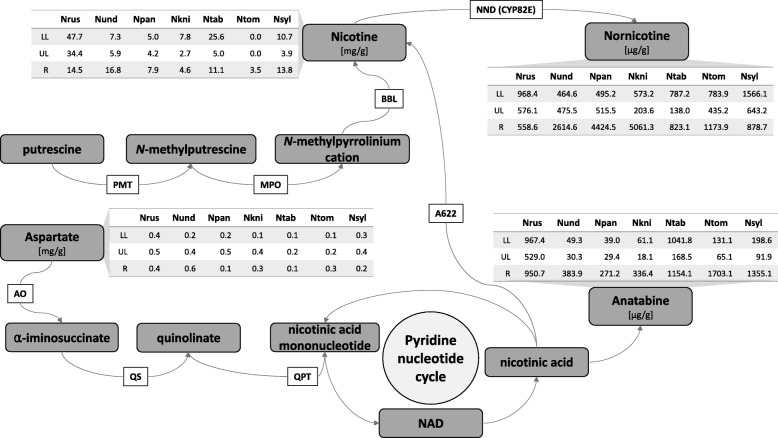
Alkaloid synthesis pathway with the measured metabolite contents in three different organs (LL: lower leaf, UL: upper leaf, R: root) from field-grown plants. A622, an enzyme proposed to form a coupling-competent nicotinic acid intermediate; AO, L-aspartate oxidase; BBL, berberine bridge enzyme-like protein; MPO, methylputrescine oxidase; NAD, nicotinamide adenine dinucleotide; NND, nicotine N-demethylase; PMT, putrecine N-methyltransferase; QPT, quinolinate phosphoribosyltransferase; QS, quinolinate synthase

**Fig. 4:**
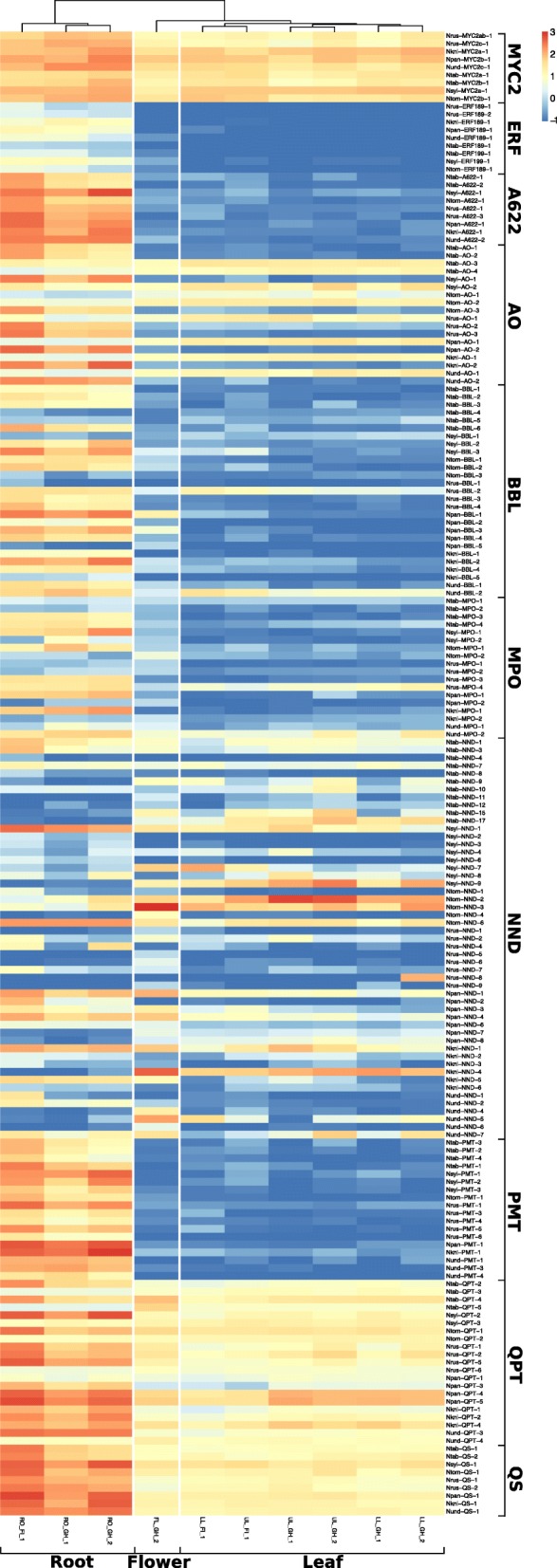
Expression of genes and key regulators of the alkaloid pathway. Log10 transformed FPKM gene expression values were used for the heatmap. LL: lower leaf, UL: upper leaf, RO: root, FL: flower, GH_1: greenhouse pre-flowering, FI: field pre-flowering, and GH_2: greenhouse flowering

The alkaloid pattern was species dependent and consistent among the biological replicates of plants cultivated in greenhouse and field (Table 2). Under all conditions, *N. rustica* contained more nicotine in the upper and lower leaves compared with all of the other species. *N. paniculata*, *N. knightiana* and, to some extent, *N. undulata* showed high nicotine to nornicotine conversion rates in the roots, which was not the case for *N. rustica*. However, *N. rustica* contained a higher level of anatabine compared with its progenitors. In Nicotiana species, the core of the alkaloid pathway consists of 8 enzymes leading to the biosynthesis of nicotine and nornicotine. Starting from putrescine, putrescine N-methyltransferase (PMT) and N-methylputrescine oxidase (MPO) are responsible for the formation of the pyrrolidine ring of nicotine, while the pyridine ring if formed from aspartate by aspartate oxidase (AO), quinolinate synthase (QS) and quinolinate phosphoribosyl transferase (QPT). A622 and berberine bridge enzyme-like (BBL) oxidoreductases are then involved in the coupling of the two rings to form nicotine. Nicotine is further demethylated by nicotine N-demethylase (NND/CYP82E) to nornicotine. The jasmonate-inducible ERF189 and ERF199 factors are key regulators of the nicotine biosynthesis pathway in *N. tabacum*. The jasmonate signaling bHLH-family transcription factor MYC2, regulates the nicotine pathway genes by interacting with ERF189 and ERF199, and by directly binding to G box elements found in their promoters [28]. The alkaloid levels are shown in Fig. 3 in the context of the alkaloid pathway, and the expression of the alkaloid pathway genes and key regulators at the different sampling times and in the different tissues are shown in Fig. 4.

**Table 2:** Akaloid concentration from plants grown in the field, young plants grown in the greenhouse, and flowering plants grown in the greenhouse

		Nicotine [mg/g]		Nornicotine [μg/g]		Anatabine [μg/g]		Nitrate [mg/g]		Asn [mg/g]		Gln [mg/g]		Asp [mg/g]		Glu [mg/g]	
		mean	stdev	mean	stdev	mean	stdev	mean	stdev	mean	stdev	mean	stdev	mean	stdev	mean	stdev
Field																	
*N. rustica*	Lower leaf	47.7	9.2	968.4	357.1	967.4	116.0	7.9	11.2	0.2	0.1	0.8	0.4	0.4	0.1	0.9	0.3
	Upper leaf	34.4	15.5	576.1	276.9	529.0	263.8	3.5	2.7	0.3	0.2	2.0	1.5	0.5	0.1	1.1	0.3
	Root	14.5	1.4	558.6	47.0	950.7	101.6	6.5	1.0	0.4	0.1	1.2	0.4	0.4	0.1	1.1	0.2
*N. paniculata*	Lower leaf	5.0	0.6	495.2	56.1	39.0	3.9	8.9	3.4	0.2	0.1	1.4	0.3	0.2	0.0	0.5	0.1
	Upper leaf	4.2	1.0	515.5	162.9	29.4	6.2	4.9	2.6	0.4	0.1	4.8	1.6	0.5	0.2	0.6	0.1
	Root	7.9	1.7	4424.5	1238.4	271.2	81.7	4.5	0.9	0.4	0.1	1.5	0.3	0.1	0.0	1.0	0.2
*N. knightiana*	Lower leaf	7.8	1.8	573.2	175.2	61.1	9.2	4.4	3.8	0.1	0.1	0.7	0.4	0.1	0.1	0.4	0.1
	Upper leaf	2.7	0.4	203.6	17.2	18.1	3.2	1.6	1.0	0.2	0.1	1.6	0.6	0.4	0.1	1.0	0.2
	Root	4.6	1.3	5061.3	1487.9	336.4	61.1	5.3	1.7	0.7	0.3	2.1	0.3	0.3	0.2	1.4	0.2
*N. undulata*	Lower leaf	7.3	1.2	464.6	151.1	49.3	10.8	23.5	11.9	0.1	0.1	0.5	0.3	0.2	0.1	0.7	0.3
	Upper leaf	5.9	1.0	475.5	107.5	30.3	6.3	9.7	9.6	0.3	0.1	1.9	0.5	0.4	0.2	0.7	0.3
	Root	16.8	2.2	2614.6	435.3	383.9	22.9	11.9	4.0	0.4	0.1	3.0	0.6	0.6	0.2	1.6	0.4
*N. tabacum*	Lower leaf	25.6	5.7	787.2	217.5	1041.8	271.0	1.3	1.0	0.1	0.0	0.6	0.4	0.1	0.1	0.6	0.1
	Upper leaf	5.0	0.4	138.0	29.5	168.5	29.5	2.7	1.7	0.5	0.3	4.4	2.3	0.2	0.1	1.2	0.5
	Root	11.1	1.5	823.1	130.2	1154.1	168.4	4.6	3.2	0.2	0.1	1.4	0.3	0.1	0.0	0.7	0.2
*N. sylvestris*	Lower leaf	10.7	1.9	1566.1	970.3	198.6	25.3	7.7	7.6	1.3	1.6	2.3	2.2	0.3	0.2	0.4	0.1
	Upper leaf	3.9	1.2	643.2	496.0	91.9	49.5	3.7	3.2	3.3	3.0	4.8	3.9	0.4	0.3	0.7	0.2
	Root	13.8	1.7	878.7	130.0	1355.1	320.8	8.8	2.5	0.8	0.4	2.1	0.7	0.2	0.1	0.8	0.3
*N. tomentosiformis*	Lower leaf	0.0	0.0	783.9	96.8	131.1	24.3	0.8	1.0	0.2	0.1	0.8	0.2	0.1	0.0	0.5	0.2
	Upper leaf	0.0	0.0	435.2	66.1	65.1	17.1	2.0	2.2	0.3	0.1	0.8	0.4	0.2	0.1	0.8	0.2
	Root	3.5	0.3	1173.9	161.7	1703.1	62.0	6.8	3.6	0.5	0.2	1.8	0.3	0.3	0.1	1.3	0.2
Greenhouse -- young plants																	
*N. rustica*	Lower leaf	15.1	2.1	201.8	46.9	235.5	117.0	31.1	1.7	0.3	0.1	0.8	0.1	0.8	0.2	1.6	0.2
	Upper leaf	27.0	5.0	304.9	41.4	173.4	44.1	13.6	4.3	0.6	0.1	2.2	0.6	1.2	0.1	2.1	0.2
	Root	7.2	1.0	240.4	41.0	421.3	122.0	11.3	3.0	0.3	0.0	0.5	0.0	2.0	0.2	0.8	0.1
*N. paniculata*	Lower leaf	4.5	0.4	702.4	220.7	92.2	122.6	52.4	19.2	0.3	0.2	2.0	0.6	0.6	0.2	1.4	0.3
	Upper leaf	4.7	1.1	629.4	152.5	11.3	9.3	23.4	9.5	0.2	0.1	3.4	1.3	1.1	0.4	2.2	0.4
	Root	5.5	0.9	3543.3	1117.7	67.5	43.1	10.3	2.7	0.2	0.1	0.2	0.0	1.2	0.3	0.8	0.1
*N. knightiana*	Lower leaf	8.2	2.3	430.4	155.6	66.4	20.6	9.3	6.4	0.1	0.0	1.1	0.2	0.5	0.1	1.1	0.2
	Upper leaf	7.6	3.1	536.8	244.9	19.3	9.4	3.6	2.8	0.1	0.0	1.3	0.3	0.7	0.1	2.0	0.2
	Root	6.7	0.9	2861.5	783.4	155.9	15.5	5.1	1.4	0.6	0.2	0.2	0.0	2.7	0.2	0.9	0.1
*N. undulata*	Lower leaf	5.0	2.1	84.3	37.4	52.6	27.2	74.0	33.3	0.1	0.0	1.8	1.2	0.3	0.1	1.0	0.3
	Upper leaf	10.6	6.1	187.4	96.2	78.1	32.3	20.7	7.6	2.2	3.0	3.8	0.9	0.9	0.1	2.6	0.8
	Root	5.6	2.2	682.1	230.8	371.6	180.9	12.2	2.0	0.1	0.0	0.4	0.1	1.9	1.1	1.0	0.3
*N. tabacum*	Lower leaf	10.8	2.1	289.0	64.9	350.8	44.3	1.0	0.7	0.1	0.0	0.6	0.1	0.3	0.0	1.5	0.2
	Upper leaf	6.1	1.2	166.6	29.9	129.4	31.9	2.5	0.6	0.2	0.0	3.5	0.4	0.9	0.1	2.0	0.2
	Root	4.0	0.4	244.7	34.4	292.2	21.2	2.8	0.4	0.1	0.0	0.2	0.0	1.4	0.3	0.5	0.1
*N. sylvestris*	Lower leaf	6.3	1.3	225.6	154.5	35.6	9.3	7.8	5.9	0.1	0.0	0.8	0.6	0.2	0.1	0.6	0.2
	Upper leaf	4.7	0.8	128.5	40.1	34.2	7.4	6.1	2.1	0.1	0.0	2.5	0.5	0.7	0.2	1.3	0.2
	Root	8.1	1.4	777.7	218.9	360.5	32.0	4.0	1.3	0.1	0.0	0.3	0.1	1.5	0.5	0.8	0.1
*N. tomentosiformis*	Lower leaf	0.0	0.0	770.2	176.8	140.4	30.3	1.2	1.9	0.2	0.0	1.3	0.4	0.6	0.1	1.3	0.4
	Upper leaf	0.0	0.0	450.1	159.0	77.0	25.5	2.5	1.6	0.4	0.1	5.3	0.9	1.4	0.2	1.6	0.2
	Root	1.7	0.5	352.2	101.6	639.2	129.3	6.2	1.7	0.7	0.2	0.5	0.1	2.4	0.4	1.0	0.2
Greenhouse -- flowering plants																	
*N. rustica*	Lower leaf	24.7	8.6	400.6	418.9	571.5	338.2	34.7	6.0	0.6	1.0	1.2	0.5	0.6	0.4	1.9	0.8
	Upper leaf	30.5	9.5	448.5	72.8	642.6	417.4	32.7	7.2	0.4	0.5	1.5	0.2	0.7	0.3	1.4	0.4
	Root	7.8	2.5	313.9	99.4	514.4	109.1	9.9	4.7	0.5	0.3	0.5	0.1	1.4	0.6	0.8	0.2
	Flower	5.8	4.6	193.8	41.9	68.3	44.9	1.1	0.5	1.6	1.3	1.3	0.3	0.4	0.0	0.7	0.1
*N. paniculata*	Lower leaf	6.4	3.7	747.6	449.5	134.4	204.9	50.7	18.6	0.1	0.0	1.1	0.5	0.2	0.1	0.7	0.4
	Upper leaf	2.0	0.8	221.7	88.9	0.0	0.0	38.0	8.9	0.3	0.2	3.2	2.1	1.5	0.7	2.3	0.3
	Root	5.4	2.2	3620.3	1363.9	67.0	35.5	4.9	1.3	0.2	0.1	0.1	0.0	1.0	0.3	0.5	0.1
	Flower	1.8	0.8	162.5	78.3	0.0	0.0	2.0	0.8	2.5	0.7	6.6	2.8	0.5	0.1	1.4	0.2
* N. knightiana*	Lower leaf	6.8	0.9	264.7	76.4	122.7	15.3	10.1	3.8	0.0	0.0	0.3	0.0	0.2	0.1	1.3	0.5
	Upper leaf	4.4	1.1	314.3	95.7	30.9	38.9	13.3	4.1	0.2	0.1	1.0	0.1	1.4	0.5	2.0	0.5
	Root	7.5	1.8	5199.8	1522.4	289.1	117.1	5.8	1.4	1.0	0.5	0.2	0.2	2.2	0.7	0.7	0.3
	Flower	0.9	0.4	26.7	10.3	0.0	0.0	0.8	0.3	1.7	0.4	5.0	1.8	0.5	0.2	1.2	0.3
*N. undulata*	Lower leaf	16.3	5.7	650.0	207.5	258.9	174.3	18.9	12.2	0.1	0.1	0.6	0.2	0.5	0.1	1.0	0.1
	Upper leaf	9.4	0.3	702.9	360.1	77.3	48.7	6.5	1.7	0.0	0.0	0.4	0.0	0.6	0.1	0.9	0.1
	Root	7.6	2.3	2112.3	1042.3	379.9	212.2	17.4	2.4	0.6	0.0	0.8	0.3	3.0	0.6	1.0	0.4
	Flower	0.3	0.1	70.3	5.4	0.0	0.0	0.3	0.1	1.9	0.5	8.3	2.4	1.6	0.2	1.1	0.4
*N. tabacum*	Lower leaf	10.0	2.6	323.5	67.6	429.9	90.2	1.7	0.9	0.1	0.2	0.4	0.1	0.4	0.1	1.1	0.2
	Upper leaf	8.2	1.9	251.5	79.4	290.1	77.7	3.9	2.0	0.1	0.0	0.8	0.1	0.9	0.1	2.2	0.6
	Root	4.8	0.4	425.1	53.6	380.9	34.0	1.6	0.4	0.3	0.1	0.1	0.0	1.3	0.3	0.3	0.1
	Flower	2.1	0.3	65.6	29.1	36.5	8.3	7.5	1.2	10.7	1.8	14.5	3.2	1.2	0.1	1.4	0.2
*N. sylvestris*	Lower leaf	4.1	0.3	178.3	38.1	31.0	10.1	12.6	6.3	0.1	0.1	0.3	0.1	0.2	0.1	0.5	0.3
	Upper leaf	2.6	0.3	79.8	25.6	45.2	17.1	19.2	9.5	0.1	0.0	0.4	0.1	0.5	0.1	0.6	0.1
	Root	7.4	1.4	653.8	184.9	726.2	132.6	5.2	1.5	0.9	0.1	0.4	0.1	4.4	0.9	0.6	0.1
	Flower	0.2	0.2	5.9	4.3	0.0	0.0	3.0	0.7	2.6	0.4	7.2	0.5	0.7	0.1	0.6	0.1
*N. tomentosiformis*	Lower leaf	0.0	0.0	0.0	0.0	331.2	135.1	7.0	2.1	0.7	0.7	0.7	0.2	1.2	0.3	1.2	0.3
	Upper leaf	0.0	0.0	0.0	0.0	129.2	66.5	14.7	3.6	1.5	0.9	1.9	0.5	1.8	0.5	1.6	0.3
	Root	0.8	0.3	1719.7	763.5	849.9	224.8	9.2	2.0	7.0	1.0	0.6	0.2	9.5	1.9	1.1	0.3
	Flower	0.0	0.0	0.0	0.0	4.8	0.0	1.1	0.6	9.8	3.5	9.3	2.7	1.1	0.1	2.3	0.1

For each enzyme of the alkaloid pathway, the copy numbers of the encoding genes and their expression levels in the various organs were analyzed in *N. rustica*, *N. tabacum* and their respective ancestors to identify changes, which could explain differences in alkaloid accumulations. An additional putrescine methyltransferase (*PMT*) gene was identified in the *N. rustica* genome in comparison with the *N. tabacum* genome (Additional file 5: Figure S4a). From the six *PMT* genes identified in *N. rustica*, four (*PMT-2*, *-3*, *-5* and -*6*) have homologs in *N. undulata* (*PMT-1*, -*2*, -*3* and -*4*, respectively), and the two others (*PMT-1* and -*4*) likely originated from a single gene in *N. knightiana*/*N. paniculata* (*PMT-1*). Interestingly, the PMT sequences from *N. rustica* and their corresponding progenitors, except PMT-2, slightly diverged from *N. tabacum* sequences, as shown in Additional file 5: Figure S4a. In comparison, only four *PMT* genes were identified in *N. tabacum* as published by Riechers and Timko [29] and deposited in the Uniprot database. Three (*PMT-2*, -*3* and -*4*) have homologs in *N. sylvestris* (*PMT-1*, -*2* and -*3*), and one (*PMT-1*) in *N. tomentosiformis* (*PMT-1*, based on intron and promoter sequence comparisons; data not shown). In *N. tomentosiformis*, a second copy close to *PMT-1* is present (*PMT-2*). In the *N. tabacum* genome, an additional small *PMT* fragment was also found, likely originating from the *N. tomentosiformis PMT-1* copy.

At the transcriptional level, the *PMT* genes were expressed almost exclusively in roots [29]. In *N. rustica*, only five *PMT* genes (*PMT-1*, -*3*, -*4*, -*5* and -*6*) were expressed under our experimental conditions, while *PMT-2* and its homolog in *N. undulata* (*PMT-2*) were silent.

In *N. tabacum*, the four *PMT* genes (*PMT-1*, -*2*, -*3* and -*4*) were expressed. In *N. tomentosiformis*, the second copy (*PMT-2*) was not expressed under our experimental conditions. This lack of expression could result from the two-fold reduction of putative binding sites for transcription factors in the promoter of *PMT-2* (based on JASPAR predictions) [30]. Because *PMT* is a key gene in nicotine metabolism [31], the addition of one more expressed gene in *N. rustica* compared with *N. tabacum* may increase nicotine biosynthesis in *N. rustica*. Based on the data presented in Additional file 5: Figure S4a, we hypothesize that a main difference between *N. rustica* and its progenitors is the root-to-shoot transport of nicotine.

### Nornicotine biosynthesis

Nornicotine is generated through the N-demethylation of nicotine, which involves cytochrome P450 nicotine N-demethylases (NNDs). In tobacco leaves, three active enzymes, CYP82E4, CYP82E5 and CYP82E10, have been identified [32]. As shown in Fig. 3, the nornicotine content may vary between *N. rustica* and its progenitors, as well as between *N. tabacum* and its progenitors. The nornicotine content is particularly abundant in the roots of *N. rustica* progenitors as compared with that in the lower leaves of *N. rustica* (five to nine times more elevated) and with *N. sylvestris* (two times more abundant compared with upper leaves and roots) (Fig. 3). To determine whether a correlation exists between gene expression and the nornicotine content, a phylogenetic tree based on sequences and sequence fragments identified in the genomes was generated. A large number of translated sequences encoding *CYP82E* genes, nine in *N. rustica*, eight in *N. paniculata*, six in *N. knightiana*, seven in *N. undulata*, 17 in *N. tabacum*, 10 in *N. sylvestris* and seven in *N. tomentosiformis*, were found. Nornicotine is approximately five, eight and nine times more elevated in the roots of *N. undulata*, *N. paniculata* and *N. knightiana*, respectively, than in the roots of *N. rustica*. This is in agreement with the reduced expression levels of the two *N. rustica NND* genes, *NND2* (10.25 fragments/kb/million mapped reads [FPKM]) and *NND7* (10.53 FPKM), compared with their corresponding progenitor genes in *N. paniculata*, *NND1* (166.88 FPKM) and *NND4* (127.4 FPKM), *N. knightiana*, *NND1* (145.69 FPKM), and *N. undulata*, *NND7* (33.01 FPKM). Furthermore, *N. undulata* and *N. paniculata* also have one more expressed gene each, *NND1* (33.88 FPKM) and *NND2* (96.85 FPKM), respectively, which are not present in *N. rustica*. The observations made at the transcriptional level were valid under field and greenhouse conditions. Thus, both the reduced expression levels of the *N. rustica NND* accessions compared with the corresponding progenitor accessions, and the presence of additional transcripts in *N. rustica* progenitors may contribute to the elevated nornicotine concentrations in the roots.

In *N. sylvestris*, nornicotine is approximately two and five times more abundant than in *N. tabacum* in the lower and upper leaves, respectively (Fig. 3). This observation was correlated with the higher transcript levels of *N. sylvestris NND7* (*CYP82E2*), *NND1* (*CYP82E10*) and *NND9* compared with the corresponding genes in *N. tabacum*, *NND9* (*CYP82E2*), *NND3* (*CYP82E10*) and *NND17*, respectively (Additional file 5: Figure S4b). We have to consider as well that mutations play a role for the activity of NNDs, as demonstrated for CYP82E2 and CYP82E3 not being active in *N. tabacum* [32].

### Leaf nicotine accumulation

**Fig. 5:**
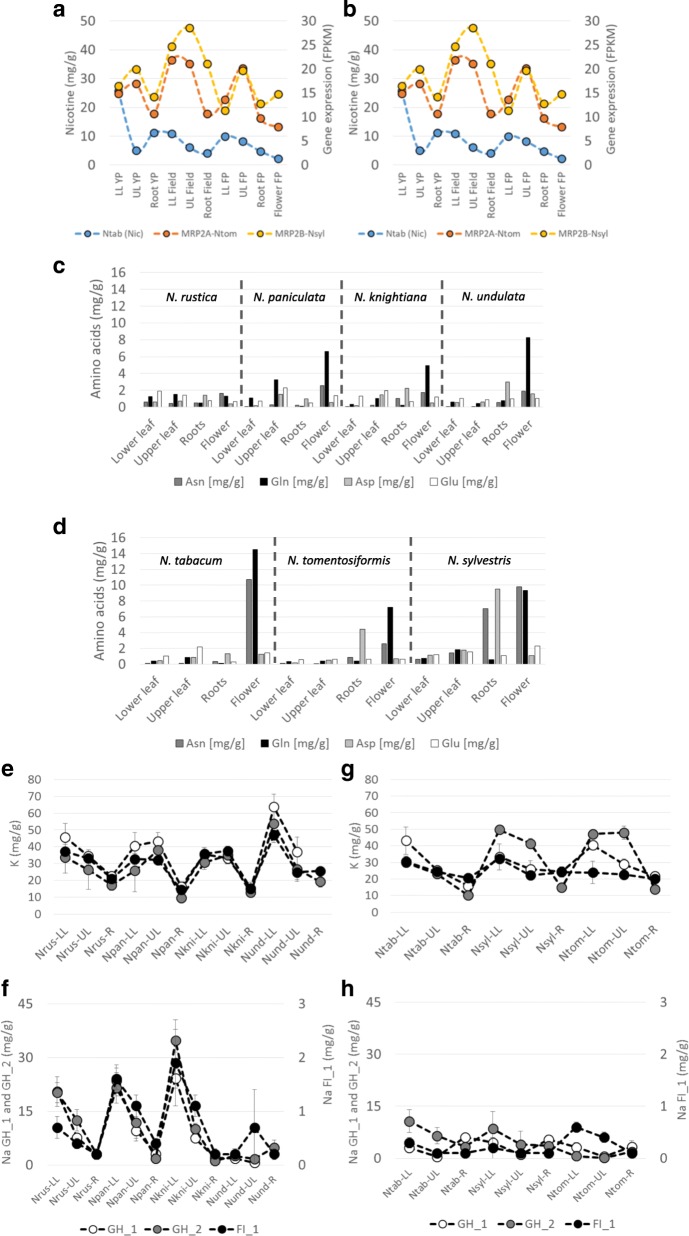
Profile alignments of the nicotine content and *MRP2A* and *MRP2B* transcripts in *N. rustica* (Nrus) (**a**) and *N. tabacum* TN90 (**b**) in the roots (Root YP), and lower (LL YP) and upper (UL YP) leaves of young plants grown in the greenhouse, in the roots (Root Field), lower (LL Field) and upper (UL Field) leaves of plants grown in the field, and finally in the roots (Root FP), flower (Flower FP) and lower (LL FP) and upper (UL FP) leaves of flowering plants grown in the greenhouse. Profiles of asparagine (Asn), glutamine (Gln), aspartate (Asp) and glutamate (Glu) contents (mg/g DW) in *N. rustica* (Nrus) and its progenitors, *N. paniculata* (Npan), *N. knightiana* (Nkni) and *N. undulata* (Nund) (**c**) and *N. tabacum* (Ntab) and its progenitors, *N. tomentosiformis* (Ntom) and *N. sylvestris* (Nsyl) (**d**). Data were collected from flowering plants in the greenhouse. Sodium (Na) and potassium (K) profiles in *N. rustica* and its progenitors (**e** and **f**) as well as in *N. tabacum* and its progenitors (**g** and **h**). Data were collected in young plants grown in the greenhouse, but root data are missing for *N. undulata*. LL: lower leaf, UL: upper leaf, R: root, FL: flower, GH_1: greenhouse pre-flowering, FI_1: field pre-flowering and GH_2: greenhouse flowering. In graphic **a**, **b**, **f** and **h**, curves were aligned to ease comparison independently of the calculated correlations

The transport of both anatabine and nicotine from root-to-shoot increased in both *N. rustica* and *N. tabacum* compared with their progenitors, particularly for mature plants grown in the greenhouse and field (Table 2). This suggests that the improved transport of both alkaloids may result from the combination of the progenitor genome after the allotetraploid formation. Because all alkaloid transporters are not yet identified in plants, particularly the transporter(s) controlling nicotine translocation from root-to-shoot [33], we looked at genes being co-expressed with anatabine and nicotine profiles. For this purpose, we calculated the correlations between the expression levels of every gene with the alkaloid concentrations across all tissues, and selected the most highly correlated for a more detailed inspection. For anatabine, no transcripts with high correlations were found; however, the transcripts of two ABC transporters, named MRP2A and MRP2B owing to their high homology with Arabidopsis multidrug resistance-associated protein 2 (MRP2, AtABCC2), exhibited high correlations (0.96 and 0.93, respectively) with nicotine in *N. rustica* (Fig. 5). *MRP2A* is from the *N. undulata* progenitor, and *MRP2B* is from the *N. paniculata* or *N. knightiana* progenitor. In Arabidopsis, AtABCC2 (MRP2) has two different activities: (1) in association with AtABCC1, AtABCC2 confer tolerance to cadmium and mercury, in addition to their role in arsenic detoxification, possibly involving phytochelatin detoxification process [34–37] (2) this ABC transporter is also involved in vacuolar transport of chlorophyll catabolites [37].

In contrast to *N. rustica*, *MRP2A* and *MRP2B* transcript profiles were not correlated with nicotine in *N. tabacum* TN90. *MRP2A* was inherited from the *N. tomentosiformis* progenitor and *MRP2B* from the *N. sylvestris* progenitor. These data suggest that the activities of *N. rustica MRP2* genes may be triggered by the nicotine content or play a role in the nicotine root-to-shoot transport of *N. rustica* specifically. In both progenitors of the allotetraploids *N. rustica* and *N. tabacum*, *MRP2* transcript profiles exhibited no correlation with the nicotine profiles (data not shown), suggesting that the synchronization of *MRP2A* and *MRP2B* transcripts with nicotine occurred after the hybridization of parental genomes.

### Amino acid pathways involving glutamate, glutamine (Gln), aspartate and asparagine

Glutamate, Gln, aspartate and asparagine are central regulators of nitrogen assimilation, metabolism and transport. In both *N. rustica* and *N. tabacum* progenitors, Gln is abundant in the flowers of greenhouse-cultivated plants. Similarly high levels of Gln are also found in *N. tabacum* but not in *N. rustica* (Fig. 5). Gln synthesis is dependent on the glutamate synthetase (GS) activity, which plays an essential role in nitrogen metabolism. The enzyme catalyzes the condensation of glutamate and ammonia to form Gln. We identified 9 and 10 different *GS* genes in *N. rustica* and *N. tabacum*, respectively, all of which were expressed. A phylogenetic tree grouped the *GS* genes into four clusters (Additional file 5: Figure S4c), independent of the Nicotiana species, thereby indicating the importance of sequence conservation during evolution. The expression data aligned with the gene clustering, highlighting the correlation between structure and function in this gene family.

Additionally, expression data helped to identify the candidate genes responsible for the regulation of Gln formation in flowers (Cluster I). Indeed, *N. knightiana GS-1* and its homolog *N. paniculata GS-4* are more than eight times more highly expressed in flowers than the corresponding genes of *N. rustica* (*GS-3*), whereas *N. undulata GS-5* is more than four times more highly expressed than the corresponding gene of *N. rustica* (*GS-7*). However, the reason for the lower expression levels of *N. rustica GS-3* and *GS-7* than the corresponding progenitor genes remains obscure. The orthologous genes of *N. tabacum* (*GS-8* and *GS-10*, respectively) belonging to the same cluster are less downregulated (< 2.5×) than the corresponding genes in *N. tomentosiformis* (*GS-3*) and *N. sylvestris* (*GS-4*). In addition, *N. sylvestris GS-4* has an additional copy in *N. tabacum (GS-6*) which may contribute to there being more Gln in *N. tabacum* than in *N. rustica* flowers.

All of these clustered genes, which are particularly expressed in non-photosynthetic tissues, like flowers and mature roots, are cytosolic *GS1*s that are involved specifically in the remobilization of nitrogen for seed feeding [38].

The observations made above for GS are not applicable for asparagine synthetase (*ASN*, data not shown), although the amino acid data suggested strong ASN activities in roots and flowers of *N. sylvestris* and in flowers of *N. tabacum* compared with other tissues, and with *N. rustica* and *N. rustica* progenitors. This may be because asparagine synthesis depends on multiple factors, like the size of the Gln and aspartate pools, as well as the level of ATP as an energy source [39, 40], and not just gene activation.

### Metal accumulations

*N. rustica* accumulates less cadmium (Cd) in leaves than *N. tabacum* under hydroponic conditions and at relatively high Cd concentrations (1 μM) [6]. In the data presented in Additional file 2: Table S2, under field and greenhouse conditions, no major differences were found, confirming previously published data [6], suggesting that leaf Cd accumulation is different when *N.rustica* and *N.tabacum* are grown under non- or Cd contaminated soils. Therefore it is not surprising to find no significant correlation between nicotine and cadmium in our dataset, soil used in the depicted experiments being low in Cd. However, this doesn’t exclude that Cd may interfere with nicotine synthesis or transport in high Cd contaminated soils or artificial nutrient solutions, therefore possibly involving homologous *AtABCC2* genes [34–37]. We also determined the concentrations of other metals and ions. For instance, *N. rustica* accumulated more arsenic in the root compared to *N. tabacum*, particularly under greenhouse condition. This observation is possibly correlated with the accumulation of sulfur in *N. rustica* and its ancestor *N. knightiana*, sulfur being known to play a role in the arsenic detoxification via the production of glutathione and glutathione-S-transferase [41] or even phytochelatins. In this respect, ABCC2 homologs are also possibly involved in some detoxification process [35]. The data on potassium (K) and sodium (Na) levels showed that, independent of the environmental growth conditions, the accumulation profiles in both roots and leaves were similar under greenhouse and field conditions in *N. rustica* and *N. rustica* progenitors, and much higher than in *N. tabacum* and its progenitors. This suggests that in *N. rustica* the maintenance of K and Na homeostases is robust and crucial for growth. However, this observation is less valid for *N. tabacum* and its progenitors, which showed more variation in K in greenhouse flowering leaves (GH_2, Fig. 5), and for Na in *N. tomentosiformis* in field leaves (Ntom-UL and Ntom-LL, Fig. 5), suggesting that K and Na may be subjected to more plasticity in *N. tabacum* and its progenitors.

## Discussion

Following the publication of the genome of *N. tabacum* and its progenitors, a second, independent, set of genomes containing a tetraploid Nicotiana species, *N. rustica,* and its progenitors is available to investigate the impact of speciation by hybridization of two diploid species. Unlike in *N. tabacum*, no intergenomic translocations were observed in *N. rustica*. Those present in *N. tabacum* are hypothesized to be the result of the wider divergence between the parental genomes of *N. sylvestris* and *N. tomentosiformis* compared with those of *N. rustica*, which has relatively close parental genomes from *N. undulata* and Nicotiana section Paniculata. It is possible that the similar parental genomes of *N. rustica* exert a lower “genomic stress” [18], resulting in less selection pressure and, therefore, less intergenomic translocations. Despite not giving a final answer regarding the sequence of evolutionary events leading to the speciation of *N. rustica*, *N. paniculata* and *N. knightiana*, the genome sequences of the progenitors of *N. rustica* also indicate that *N. knightiana* is more closely related to *N. rustica* than *N. paniculata*.

Focusing on the mechanism behind the upregulation of nicotine production in *N. rustica* provided insights into the metabolic and genomic differences in comparison with *N. tabacum* and its progenitors. Compared with its progenitors and with *N. tabacum* and its progenitors, *N. rustica* contained more nicotine in the upper and lower leaves. Our data suggests that nicotine level in *N. rustica* results more of a genome combination of the ancestors than in *N. tabacum*, *N. tomentosiformis* exhibiting rather low nicotine in both root and leaves compared to *N. sylvestris*. Interestingly, *N. sylvestris* has three active copies of *PMT*, one of the key root expressed gene involved in nicotine synthesis, whereas *N. tomentosiformis* has only one. Therefore, four active *PMTs* were identified in *N.tabacum*, and five in *N. rustica*, for whom the genetic origin of the ancestors is more complex to draw. In addition to more active synthesis, the elevated nicotine content in *N. rustica* compared to *N. tabacum* may also results from a more active transport to the shoot via an ABC transporter, its expression being correlated with nicotine in *N. rustica*. The nicotine to nornicotine conversion rates in the roots of *N. paniculata*, *N. knightiana* and, to some extent, *N. undulata* was high, which was not the case for *N. rustica*, which contained higher levels of anatabine compared with its progenitors. Regarding the nicotine conversion, it does not seem to derive from a simple additive gene effect, nornicotine levels being generally higher in the ancestors compared to both *N. rustica* and *N. tabacum*. This suggests some regulatory processes to occur at the *CYP82E* transcript level. Based on our data, no interconnection between Cd uptake and nicotine synthesis as well as shoot translocation can be established. Similar experiments should be performed using Cd contaminated soils. In this context, *ABBC2* (*MRP2*) homologous genes may play a role in interfering between nicotine and Cd accumulation, AtABCC2 carrying already different substrate affinity [34–37]. About two times more asparagine and glutamine were found in the flower of *N. tabacum* compared to *N. rustica* suggesting a more efficient remobilization of carbon and nitrogen resources, possibly supported by different glutamine synthase activities. Finally*, N. rustica* accumulates more sulphur than *N. tabacum* particularly in the above ground organs which may support arsenic and cadmium detoxification under certain growth conditions. In addition, K and Na homeostases seem to be particularly well-controlled in *N. rustica* compared to *N. tabacum* for the maintenance of growth.

## Conclusions

The comparative genome analysis of four related Nicotiana genomes showed that the tetraploid species *N. rustica* inherited about 41% of its genome from its paternal progenitor, *N. undulata*, the rest originating for its maternal progenitor, the common ancestor of *N. paniculata* and *N. knightiana*. Analysis of the genome sequences of the progenitors of *N. rustica* indicated that *N. knightiana* is more closely related to *N. rustica* than *N. paniculata*, although the sequence of evolutionary events leading to the speciation of *N. rustica* remain to be elucidated. *N. rustica* contained more nicotine in the upper and lower leaves than its progenitors, this nicotine level likely being the results of the genome combination of the progenitors. A more active transport of nicotine to the shoot via an ABC transporter in *N. rustica* may also contribute to the elevated nicotine content in *N. rustica* compared to *N. tabacum* in addition to the presence of one more additional *PMT* copy in the *N. rustica* genome. The availability of these new set of related Nicotiana genome sequences, will significantly contribute to better understanding the impact of speciation and the evolution of tetraploid Nicotiana species.

## Methods

### Plant material

*N. rustica* L. var. Brasilia No. 7 (PI 499174, TR13, USDA–GRIN database), *N. paniculata* L. (PI 555545, TW 99, USDA–GRIN database), *N. knightiana* Goodsp. (PI 555527, TW 73, USDA–GRIN database), *N. undulata* Ruiz & Pav. (PI 555575, TW 146, USDA–GRIN database), *N. tabacum* L. cv. TN90 (PI 543792, TC 586, USDA–GRIN database), *N. sylvestris* Speg. & Comes (PI 555569, TW 136, USDA–GRIN database) and *N. tomentosiformis* Goodsp. (PI 555572, TW 142, USDA–GRIN database) were used in the experiments.

### Plantlet growth and hydroponics

Seeds were sown on soil. For the field experiment, plants were transferred after 3 weeks into soil-containing floating trays (floating tray solution: Hauert Plantaaktiv 15 + 7 + 22; Hauert, Grossaffoltern, Switzerland). Plants were grown for six more weeks in hydroponics before being transferred to the field. For the greenhouse experiment, plants were directly transferred to pots.

### Greenhouse experiment

The solutions used for the fertilization of soil-grown plants were purchased from Yara Benelux B.V. (Vlaardingen, The Netherlands) and contained (per liter): 605.62 mg NO_3_, 13.29 mg NH_4_ (total of 147.77 mg N), 65.56 mg P_2_O_5_, 275.44 mg K_2_O, 35.89 mg Mg, 133.26 mg Ca, 265.35 mg SO_4_, 0.516 mg Fe, 0.338 mg Mn, 0.201 mg Zn, 0.199 mg B, 0.029 mg Cu and 0.03 mg Mo. All of the plants were grown in a 16-h light:8-h dark cycle. First time points for plant sampling took place after 10 weeks of growth. At this time point, *N. rustica* and *N. paniculata* were already flowering, and *N. knightiana* and *N. sylvestris* were starting to flower. Next, plants were sampled again at a second time point when fully flowering. *N. rustica*, *N. paniculata*, *N. knightiana* and *N. sylvestris* were sampled after 12 weeks of growth, *N. tabacum* and *N. undulata* after 14 weeks and *N. tomentosiformis* was transferred to a 9-h light:15-h dark cycle to induce flowering and then sampled after 21 weeks.

### Field trial

The field experiment was conducted in Switzerland (Vaud). Prior to the field experiment, soil samples were taken to a depth of 30 cm, and mixed and analyzed by Sol Conseil (Changins, Switzerland). Furthermore, shortly before transplanting, an additional soil sample (30-cm depth) was analyzed for nitrogen content (Service de l’agriculture, Agrilogie, Grange-Verney, Moudon, Switzerland). The soil was composed of 14.1% clay, 35.5% silt and 50.4% sand, containing 1.8% organic matter, with a pH of 8.0. The soil contained 140 mg kg^− 1^ soluble Ca and, as determined by ammonium acetate-EDTA extraction, 27.0 mg kg^− 1^ P, 157.6 mg kg^− 1^ K, 50,234 mg kg^− 1^ Ca and 361.7 mg kg^− 1^ Mg. The total N was 25.9 kg ha^− 1^, composed of 23.2 kg ha^− 1^ N-NO_3_ and 2.7 kg ha^− 1^ N-NH_4_. The field was fertilized according to tobacco cultivation practices with K_2_SO_4_ [450 kg ha^− 1^ 50% + S; Landor (Birsfelden, Switzerland)], superphosphate [239 kg ha^− 1^ 18 P-4 Mg; Landor (Birsfelden, Switzerland)] and nitrochalk [97 kg ha^− 1^ total 15.5–0-0; Yara Benelux B.V. (Vlaardingen, The Netherlands)]. During the season, precipitation was measured using a rain gauge. Temperature was obtained from the nearest weather station (MétéoSuisse). The growing season was relatively dry and hot. The temperature exceeded 30 °C on 23 days (maximum of 37.9 °C) and 144 mm of rainfall were recorded. The field was irrigated twice with a total of 45 mm of water. Plants were grown in a random design. Four plants per species were harvested 80 days after transplantation. Plants were at a different physiological stages, depending on the plant species. While *N. rustica*, *N. paniculata*, *N. knightiana* and *N. sylvestris* were flowering, *N. tabacum*, *N. undulata* and *N. tomentosiformis* were not yet flowering. *N. tomentosiformis* grew very poorly in the field. Total plants, including roots, were harvested and processed as described below (section “Plant sampling”).

### Plant sampling

Roots were separated from shoots and washed with water until clean. Roots were dried with paper and flash-frozen in liquid nitrogen. They were ground in liquid nitrogen and some of the material was used for RNA extraction. Some ground root material was lyophilized and then analyzed for alkaloid, amino acid and elemental compositions. One or more leaves positioned on the lower stalks (depending on leaf size) were harvested for each plant and leaves were cut in halves (without the midrib). One half was frozen in liquid nitrogen for RNA analysis and the other half-leaf was lyophilized and analyzed for alkaloid and elemental compositions. One or more leaves positioned on the upper stalks were similarly processed. The leaf materials for RNA extractions were ground in liquid nitrogen.

### Data analysis

Four plants were analyzed for each variety and condition. Values are means ± standard deviations of four replicate plants. When the value was below the limit of reporting, the reporting limit was used for the calculation. Owing to insufficient materials being available for collection at the first sampling time point in the greenhouse, only two *N. paniculata* root samples, three *N. sylvestris* root samples and no *N. undulata* root samples were analyzed for elemental composition. However, all of the samples were analyzed for alkaloids and amino acids.

### Elemental analysis

The elemental composition of the samples was analyzed by ALS Life Sciences (Praha, Czech Republic). Samples were homogenized and mineralized by acids and hydrogen peroxide prior to analysis (CZ_SOP_D06_02_J02 chap. 10.17.1, 10.17.2, 10.17.4, 10.17.7, 10.17.8). As, Cd, Cr, Cu, Pb, Ni and Zn were measured by mass spectrometry with inductively coupled plasma according to CZ_SOP_D06_02_002 (US EPA 200.8, CSN EN ISO 17294-2). The elemental composition always refers to plant dry weight. All other elements were measured by inductively coupled plasma atomic emission spectroscopy according to CZ_SOP_D06_02_001 (US EPA 200.7, ISO 11885).

### LC-UV/MS analysis of freeze-dried plant material

Weighed aliquots (~ 25 mg) of lyophilized and pulverized plant materials were extracted with 1.8 mL of 0.1 N HCl at 90 °C for 1 h. After centrifugation, 120 μL aliquots of the supernatants were mixed with 800 μL MeCN, 40 μL of 0.33 M sodium acetate solution; and centrifuged again in a solution of isotopically labelled internal standards (215 μg/mL K^15^NO_3_; 2.0 μg/mL nornicotine-*d*_4_; 40 μg/mL nicotine-*d*_4_; 1.0 μg/mL anatabine-*d*_4_; and 10 μg/mL asparagine-^15^*N*_2_; in MeOH). The supernatants were analyzed by LC-MS on an Ultimate 3000 UHPLC system coupled to a Q-Exactive mass spectrometer (Thermo Fisher Scientific). Chromatographic separation was performed on an Acquity UPLC BEH Amide column (1.7 μm, 150 × 2.1 mm; Waters), and the column temperature was set to 20 °C. Eluents were aqueous ammonium formate (2 mM) with added formic acid (0.25% *v*/v; eluent A) and MeCN with added formic acid (0.1% v/v; eluent B) applied as a gradient (0 min–6% A; 0.5 min–6% A; 4.0 min–60% A; 4.5 min–60% A; flow: 0.5 mL/min). The injection volume was 0.7 μL. Nitrate, nicotine, anatabine, nornicotine, glutamic acid, Gln, aspartic acid and asparagine were eluted after 1.15, 3.16, 3.23, 3.32, 4.00, 4.10, 4.16 and 4.20 min, respectively. For MS detection, electrospray ionization was applied with capillary voltages of 3.7 and 2.0 kV in positive and negative modes, respectively. The nitrate ion was detected in the negative mode, while nicotine and the amino acids were detected as [M + H]^+^ pseudomolecular ions in the positive mode. For the detection of anatabine, the *m/z* 25 fragment was used after collision-induced fragmentation of the *m/z* 158 ion in the positive mode. For quantification, the respective isotopically labeled internal standards were used for nitrate, nicotine, anatabine and asparagine. Aspartic acid, Gln and glutamic acid were quantified by external calibration.

### Genome sequencing

DNA extractions were performed on the aerial parts of one plant per variety using the Qiagen DNAeasy Plant Maxi Kit (Qiagen, Hilden, Germany). Short insert “paired-end” libraries were prepared using the Illumina TruSeq DNA Sample Preparation Kit version 2 (Illumina, San Diego, CA). Long insert “mate-pair” libraries were prepared according to the Nextera Mate Pair Library Prep Kit (Illumina, San Diego, CA). All of the libraries (Additional file 2: Table S3) were sequenced on an Illumina HiSeq-2500 using version 3 chemistry and flow-cells with runs of 2 × 100 bases. Base calling and sample demultiplexing were performed using Illumina HiSeq Control Software and the CASAVA pipeline software. For *N. rustica*, 5- and 10-kb-long read libraries were prepared and sequenced on a Pacific Biosciences RSII.

### Genome size estimation

The genome sizes were estimated using the 31-k-mer depth distribution of all paired-end sequencing libraries, as described previously. Briefly, the genome sizes were obtained by dividing the total number of 31-k-mers considered to be error-free by their most frequent depths of coverage.

### De novo genome assembly

Raw paired-end DNA reads were preprocessed with Trimmomatic (http://www.usadellab.org/cms/?page=trimmomatic) to remove sequencing adapters and low quality reads from the 5′ and 3′ ends of the reads, and to discard reads shorter than 50 bp. Raw mate-paired DNA reads were preprocessed with NxTrim (https://github.com/sequencing/NxTrim) to separate them into mate-pairs and paired-ends based on the presence of the Nextera adapter. The clean reads were then assembled into contigs using SOAPdenovo2 (http://soap.genomics.org.cn/soapdenovo.html) with a k-mer of 63 and scaffolded by increasing library size. Gaps resulting from the scaffolding were closed using GapCloser (http://soap.genomics.org.cn/soapdenovo.html), and all sequences shorter than 200 bases were discarded from the final assemblies. After closing the gaps, singletons were used as queries in a BLAST-based algorithm against the scaffolds. They were eliminated if the match level was greater than 97% to avoid artificial duplications of short sequences. Long Pacific Biosciences reads were used to further scaffold the *N. rustica* assembly.

### Repeat content estimation

The repeat contents of the genome assemblies were estimated using RepeatMasker (http://www.repeatmasker.org) with the eudicot repeat library available from the Sol Genomics Network, the TIGR Solanaceae repeat library and a RepeatScout (https://bix.ucsd.edu/repeatscout) library created using sequences of at least 150 kb from the draft genome assembly. The classification of the repeat types was performed using hits to known repeat elements achieved by a BLASTN algorithm-based search.

### Assessment of genome completeness

The completeness of the genomes was assessed using Benchmarking Universal Single-Copy Orthologs (BUSCO, http://busco.ezlab.org) with the embryophyta plant dataset consisting of 1440 universal single-copy orthologs. In addition, the numbers of unique ITAG 2.3 and TAIR 10 proteins mapping to the Nicotiana genomes were determined using BLAT (http://www.kentinformatics.com) with cutoffs of 80% coverage and 80% identity for ITAG 2.3 or 60% identify for TAIR 10 (Additional file 2: Table S4).

### Transcriptome sequencing and assembly

#### Samples

For *N. rustica*, two transcriptomes were generated. The first transcriptome used RNA-seq data from eight tissues (flower bud, flower mature, capsule mature, leaf upper, leaf middle, leaf lower, root and stem) and aimed at producing a functional coverage across the tissues. In addition, to compare *N. rustica* with its progenitors using transcriptomes generated under similar conditions, a second transcriptome was generated using three tissues at two time points under two different growing conditions. Comparable samples were generated for all three putative progenitors. The samples included roots and lower and upper leaves from pre-flowering plants grown in the greenhouse, roots and lower and upper leaves from pre-flowering plants grown in the field, and roots and lower and upper leaves and flowers from flowering plants grown in the greenhouse (Additional file 6: Figure S5). Total RNA was extracted using the RNeasy Plant Mini Kit (Qiagen, Hilden, Germany).

#### Sequencing and bioinformatics

Libraries were generated using an Illumina TruSeq Stranded Kit and were sequenced on an Illumina HiSeq 2500. Reads were demultiplexed, and Trimmomatic (version 0.32) was used to remove Illumina adapters and trailing bases with a quality cutoff of 10, trim the reads and retain only paired reads with a minimum length of 50 bp. The reads were aligned using HISAT2 (https://ccb.jhu.edu/software/hisat2/index.shtml, version 2.0.1 beta). Aligned reads were filtered to include only reads flagged as PAIRED and to exclude reads flagged as SECONDARY, QCFAIL or SUPPLEMENTARY. Reads overshooting the scaffolds were removed by a custom script. For each individual sample and tissue, the mapped reads were assembled into transcripts, and these sample-wise transcript sets were then merged into a final set of transcripts using Cuffmerge (http://cole-trapnell-lab.github.io/cufflinks). Putative peptides were extracted by identifying the longest open reading frame in the transcript (Additional file 2: Tables S5–S6).

### Expressed gene family clustering with OrthoMCL

Peptides sequences from *N. rustica*, its three putative progenitors and those of *N. tabacum* TN90 were clustered to determine the structures of the gene families using the OrthoMCL software (http://orthomcl.org). For each species, transcriptomes assembled using comparable read sets were used (three tissues/three condition set). Datasets were filtered and queried using a BLAST algorithm according to OrthoMCL requirements. OrthoMCL scripts were run, and the output was compiled into a Venn diagram using the gplots library for R.

### Phylogenetic analysis

OrthoMCL was further run using the sets described above and comparable proteomes from *N. sylvestris* and *N. tomentosiformis*, as well as tomato (ITAG 2.3, reference) as an outgroup. Core gene groups were chosen from the OrthoMCL clustering based on the criterion that each group should have two representative proteins in the cluster from the allotetraploids *N. rustica* and *N. tabacum*, and one representative from the remaining diploid species. Of the 12,401 ortholog groups, approximately half fulfilled this strict ortholog-only criterion (6315). For these clusters, the putative peptides were extracted and aligned using Muscle (http://www.drive5.com/muscle) [42]. The alignments were trimmed to exclude 3′ and 5′ gapped regions, resulting in only the core consensus being included. From this set, all of the alignments were removed, of which more than 5% of the column had gaps in any one of the sequences, resulting in 3160 alignments. The alignments were used to generate a protein sequence-based phylogeny and estimate K_a_/K_s_ rates.

*Inferring N. knightiana/N. paniculata ancestry based on shared ortholog protein groups.*

For the above clusters, phylogenetic trees were built, and the list of clusters was further filtered depending on the presence of a clear clustering in the cladogram of the gene variants to their ancestors. For example, there needs to be one *N. tabacum* protein corresponding to one *N. sylvestris*, one *N. tabacum* protein clustering with one *N. tomentosiformis* protein, one *N. rustica* protein with one *N. undulata* protein and one *N. rustica* clustering closest to *N. knightiana*/*N. paniculata*. Using the clustering information, the closest neighbor was determined for the this *N. rustica* protein. This could be *N. knightiana*, *N. paniculata* or the common ancestor of both. The number of occurrences of each assignment was counted.

## Additional files

Additional file 1: **Figure S1.** *N. rustica* with green-yellow flowers. (JPG 212 kb)

Additional file 2: **Table S1.** Numbers of NPAMBO, NPAMBE and NUNDSSP repeats in the *N. rustica*, *N. paniculata*, *N. knightiana* and *N. undulata* genomes. **Table S2.** Metal accumulations in Nicotiana species in both the greenhouse and field. **Table S3.** Sequencing libraries for the *N. rustica*, *N. paniculata*, *N. knightiana* and *N. undulata* genomes. **Table S4.** Unique proteins from other plant species that map to Nicotiana genomes. **Table S5.** Transcript length statistics. **Table S6.** Protein length statistics. (XLSX 39 kb)

Additional file 3: **Figure S2.** Benchmarking Universal Single-Copy Orthologs (BUSCO) genome completeness assessment. (PDF 224 kb)

Additional file 4: **Figure S3.** Overlap of the chloroplast genomes from the four Nicotiana species based on the numbers of common SNPs. We observed 336 SNPs unique to *N. undulata*, 8 shared between *N. rustica* and *N. undulata*, 303 SNPs shared by *N. rustica*, *N. paniculata* and *N. knightiana*, 7 by *N. rustica* and *N. paniculata*, 17 by *N. rustica* and *N. knightiana* and 11 by *N. paniculata* and *N. knightiana*. (PNG 76 kb)

Additional file 5: **Figure S4.** Phylogenic tree of the putrescine methyltransferase (**a**), cytochrome P450 nicotine N-demethylase (**b**), and glutamine synthetase (**c**) proteins of *N. rustica*, *N. tabacum* and their respective progenitors, and their gene expressions, in FPKMs, in various tissues under different growth conditions. For each gene, the relative expressions are highlighted in shades of grey. Sequences for *N. tabacum* PMT1 (Q42963), PMT2 (Q9SEH7), PMT3 (Q9SEH5), PMT4 (Q9SEH4), CYP82E1 (Q9ZWK2), CYP82E2 (Q38Q85), CYP82E3 (Q38Q84), CYP82E4 (L7Y094), CYP82E5 (A1XEH1), CYP82E8 (A1XEM0), CYP82E10 (E5G962) were obtained from Uniprot. LL: lower leaf, UL: upper leaf, RO: root, FL: flower, GH_1: greenhouse pre-flowering, FI: field pre-flowering, and GH_2: greenhouse flowering. The numbers at each node of the tree correspond to the bootstrapping confidence level of each split. The gene names are composed of the abbreviated progenitor species name, the gene symbol, and the start and stop positions of the BLAST algorithm-based match expressed as a fraction of the protein length. (PDF 485 kb)

Additional file 6: **Figure S5.** Tissues, number of biological replicates and species collected for transcriptome annotation and comparative gene expression analysis. (PDF 87 kb)

Additional file 7: **File S1.** Protein sequences of the genes and key regulators of the alkaloid pathway. (TXT 90 kb)
