# Supplementary figures and images for: The impact of genome evolution on the allotetraploid Nicotiana rustica – an intriguing story of enhanced alkaloid production
Source: BMC Genomics. 2018 Nov 29;19:855. doi: 10.1186/s12864-018-5241-5 (PMC6267829; doi:10.1186/s12864-018-5241-5)

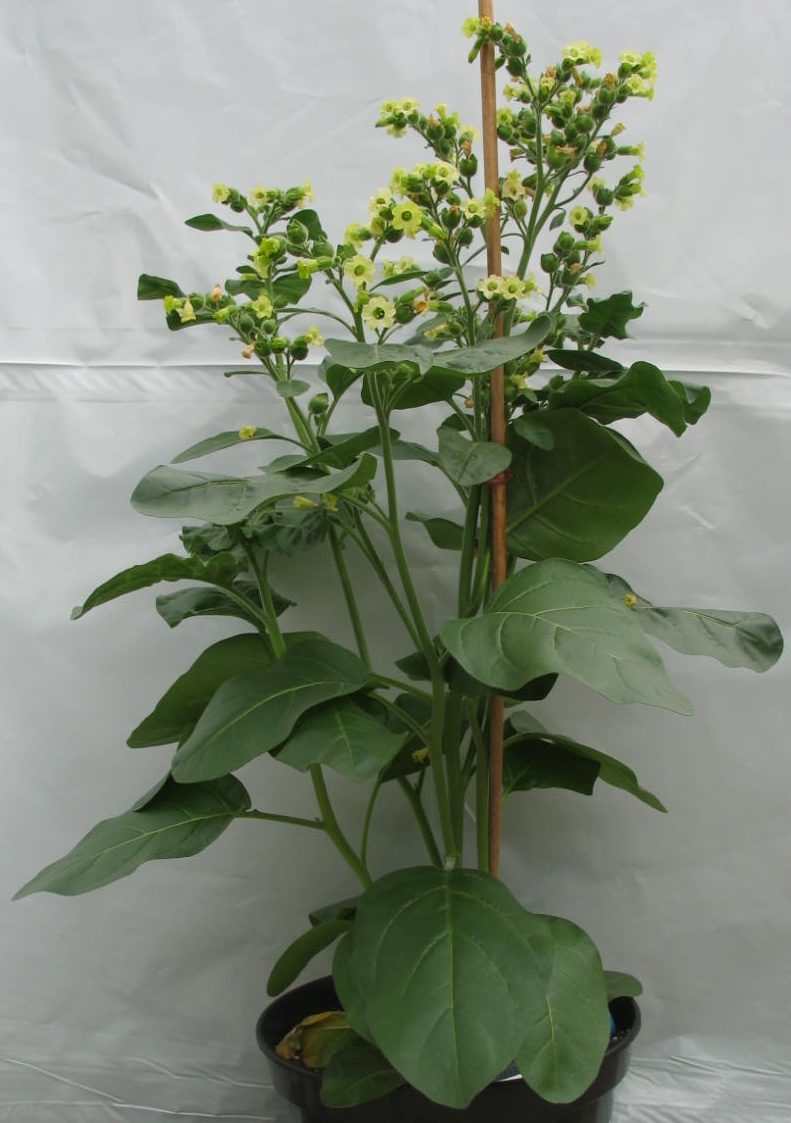

Supplement: Supplementary file 1 — Figure S1. N. rustica with green-yellow flowers. (JPG 212 kb) [file 12864_2018_5241_MOESM1_ESM.jpg]

## Genome completeness

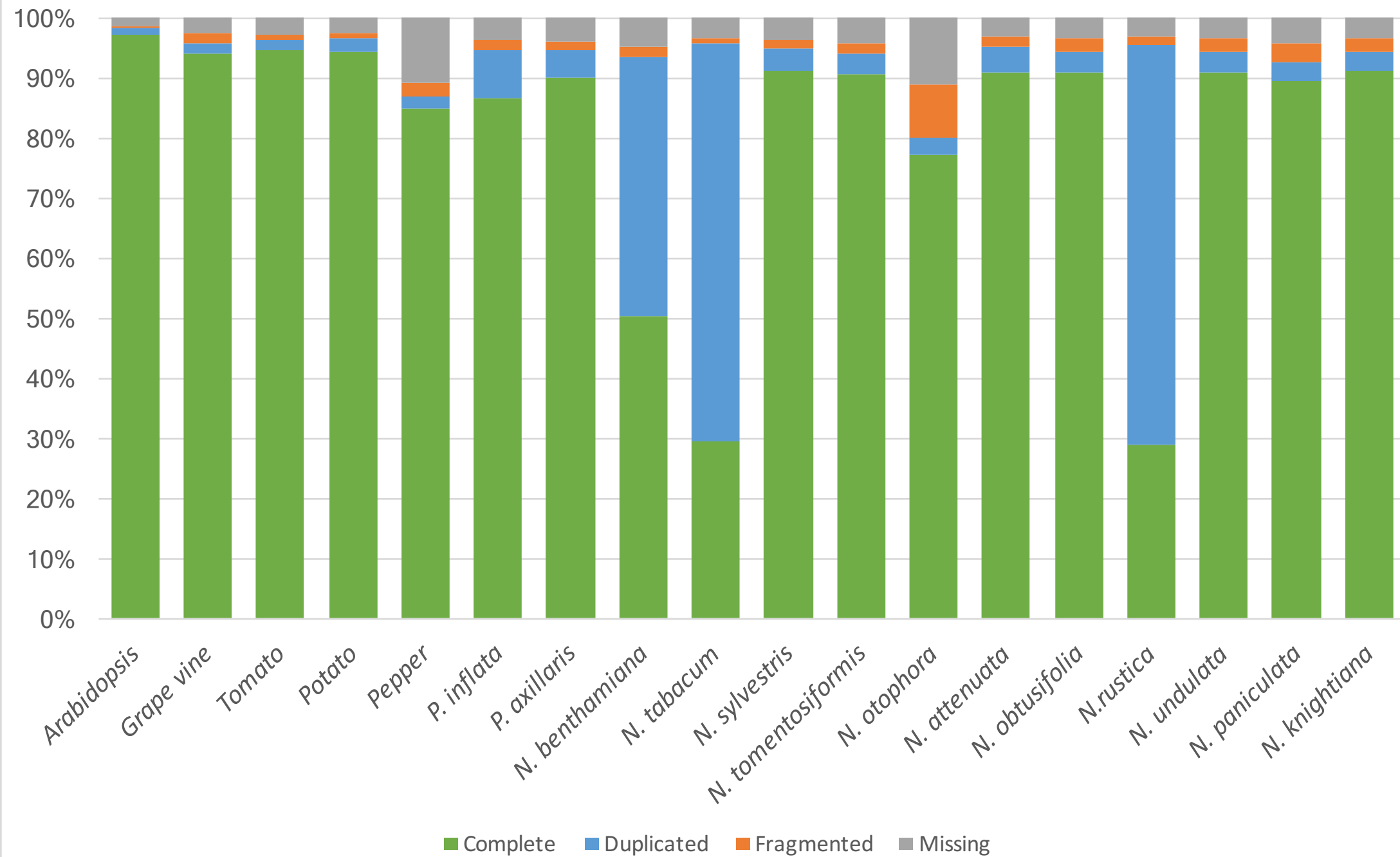

Supplement: Supplementary file 3 — Figure S2. Benchmarking Universal Single-Copy Orthologs (BUSCO) genome completeness assessment. (PDF 224 kb) [file 12864_2018_5241_MOESM3_ESM.pdf]

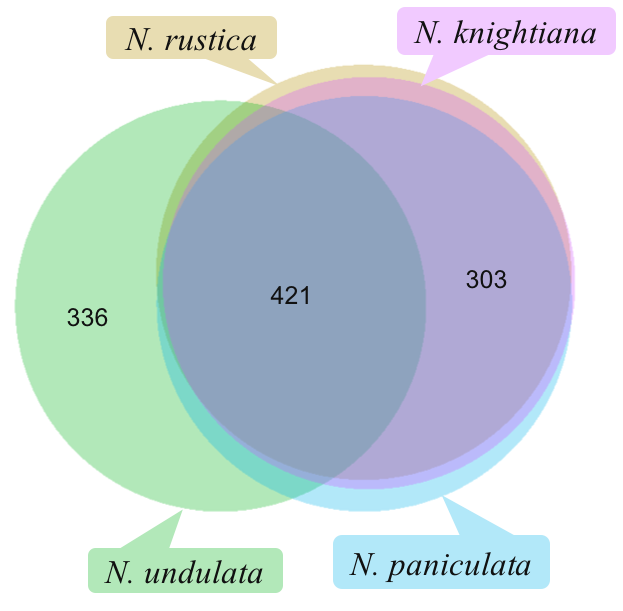

Supplement: Supplementary file 4 — Figure S3. Overlap of the chloroplast genomes from the four Nicotiana species based on the numbers of common SNPs. We observed 336 SNPs unique to N. undulata, 8 shared between N. rustica and N. undulata, 303 SNPs shared by N. rustica, N. paniculata and N. knightiana, 7 by N. rustica and N. paniculata, 17 by N. rustica and N. knightiana and 11 by N. paniculata and N. knightiana. (PNG 76 kb) [file 12864_2018_5241_MOESM4_ESM.png]

a

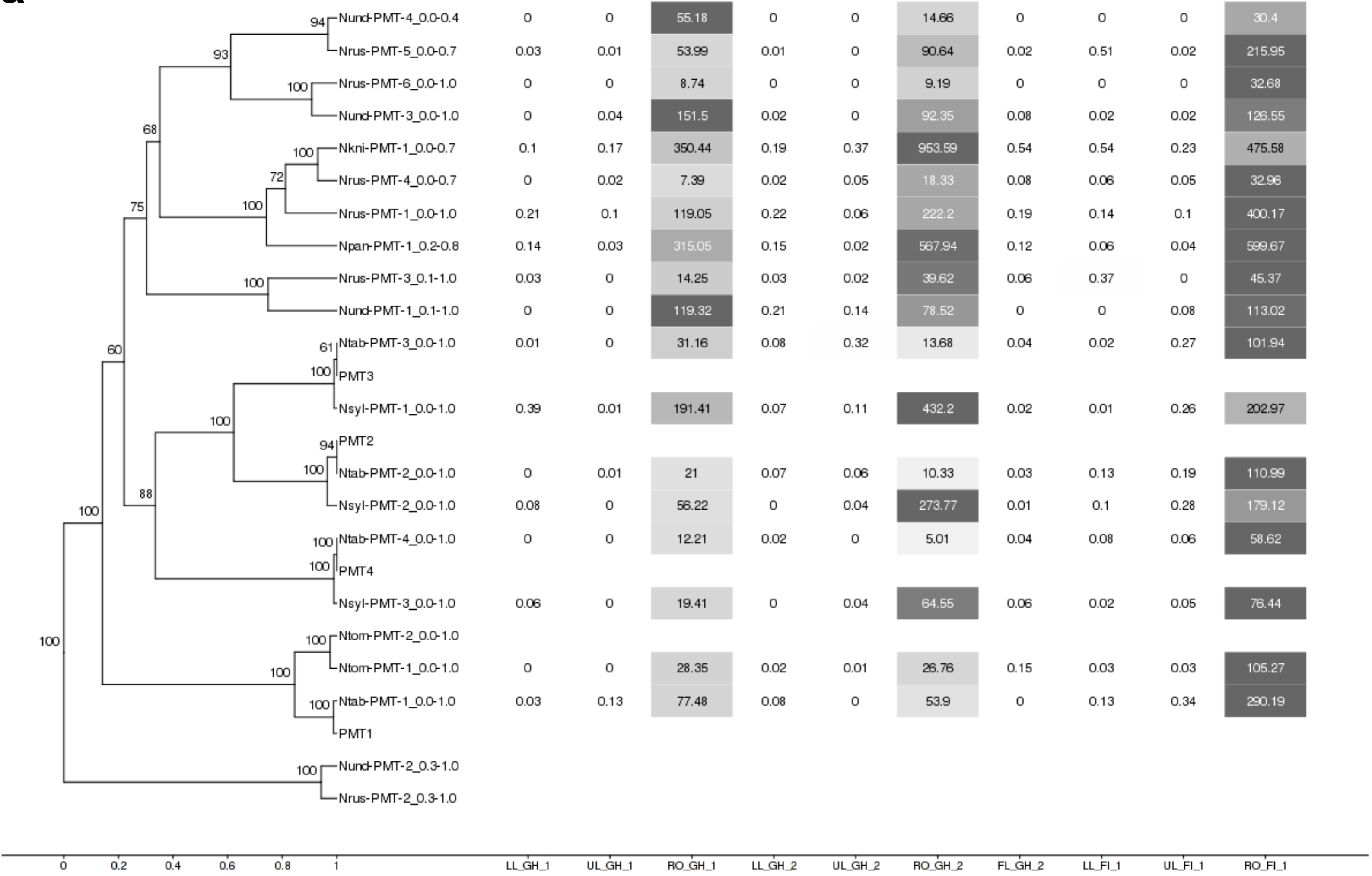

b

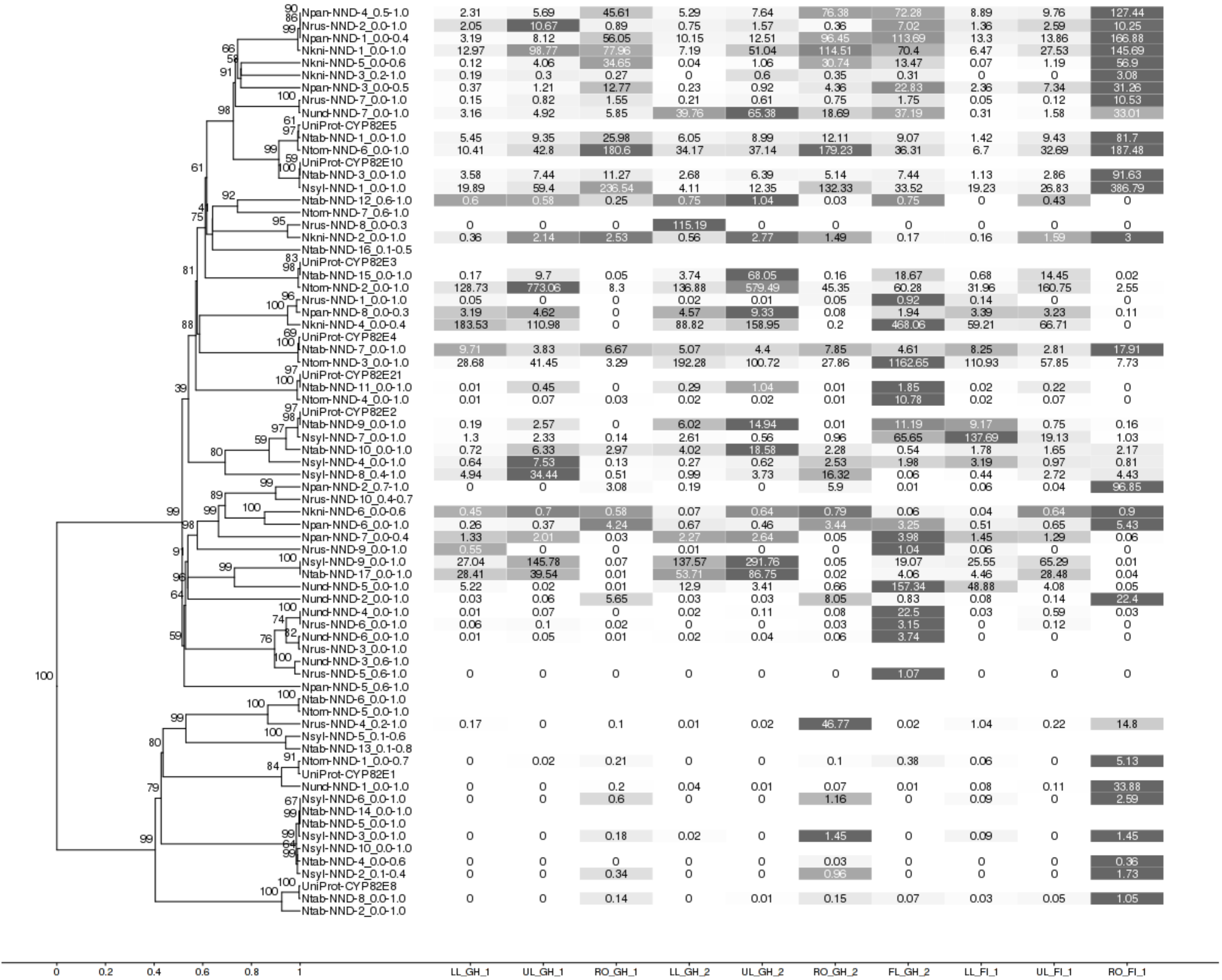

c

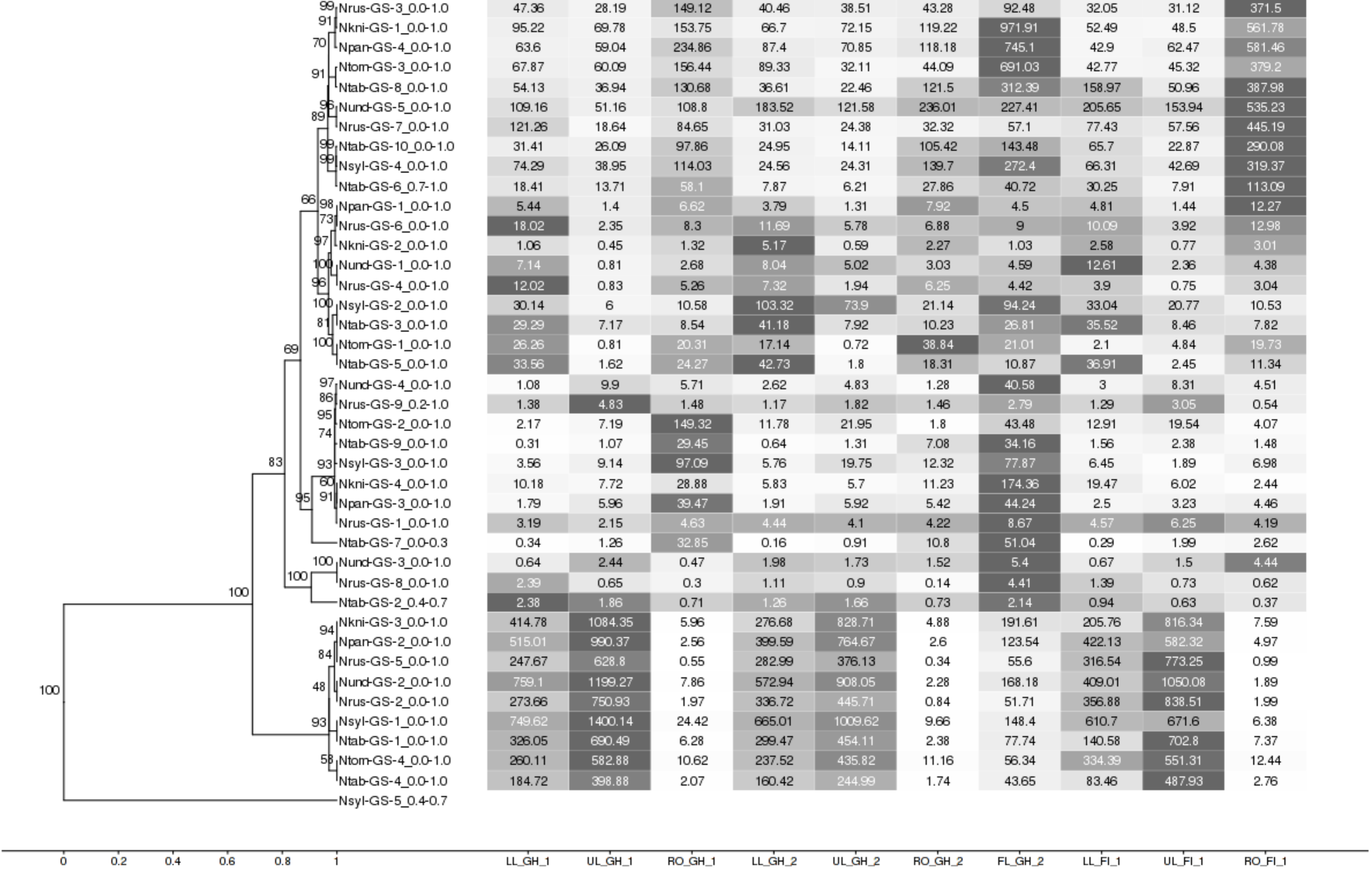

Supplement: Supplementary file 5 — Figure S4. Phylogenic tree of the putrescine methyltransferase (a), cytochrome P450 nicotine N-demethylase (b), and glutamine synthetase (c) proteins of N. rustica, N. tabacum and their respective progenitors, and their gene expressions, in FPKMs, in various tissues under different growth conditions. For each gene, the relative expressions are highlighted in shades of grey. Sequences for N. tabacum PMT1 (Q42963), PMT2 (Q9SEH7), PMT3 (Q9SEH5), PMT4 (Q9SEH4), CYP82E1 (Q9ZWK2), CYP82E2 (Q38Q85), CYP82E3 (Q38Q84), CYP82E4 (L7Y094), CYP82E5 (A1XEH1), CYP82E8 (A1XEM0), CYP82E10 (E5G962) were obtained from Uniprot. LL: lower leaf, UL: upper leaf, RO: root, FL: flower, GH_1: greenhouse pre-flowering, FI: field pre-flowering, and GH_2: greenhouse flowering. The numbers at each node of the tree correspond to the bootstrapping confidence level of each split. The gene names are composed of the abbreviated progenitor species name, the gene symbol, and the start and stop positions of the BLAST algorithm-based match expressed as a fraction of the protein length. (PDF 485 kb) [file 12864_2018_5241_MOESM5_ESM.pdf]
