# Supplementary material for: The impact of genome evolution on the allotetraploid Nicotiana rustica – an intriguing story of enhanced alkaloid production
Source: BMC Genomics. 2018 Nov 29;19:855. doi: 10.1186/s12864-018-5241-5 (PMC6267829; doi:10.1186/s12864-018-5241-5)
Supplement: Supplementary file 6 — Figure S5. Tissues, number of biological replicates and species collected for transcriptome annotation and comparative gene expression analysis. (PDF 87 kb) [file 12864_2018_5241_MOESM6_ESM.pdf]

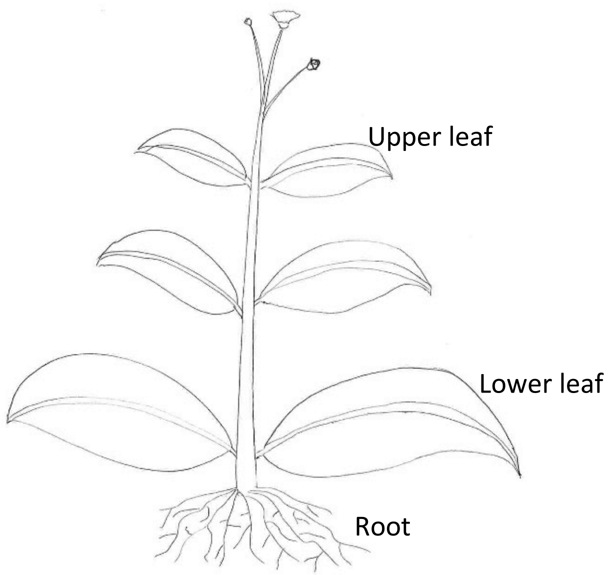

GREENHOUSE 1

4 biological replicates of

- *N. rustica*
- *N. undulata*
- *N. paniculata*
- *N. knightiana*

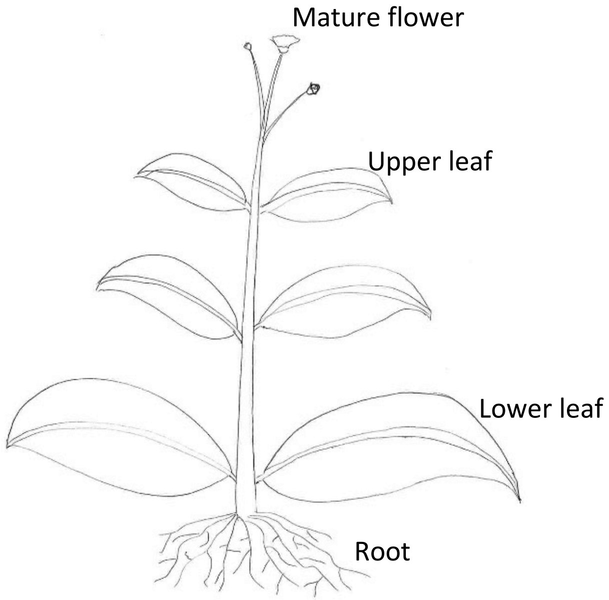

GREENHOUSE 2

4 biological replicates of

- *N. rustica*
- *N. undulata*
- *N. paniculata*
- *N. knightiana*

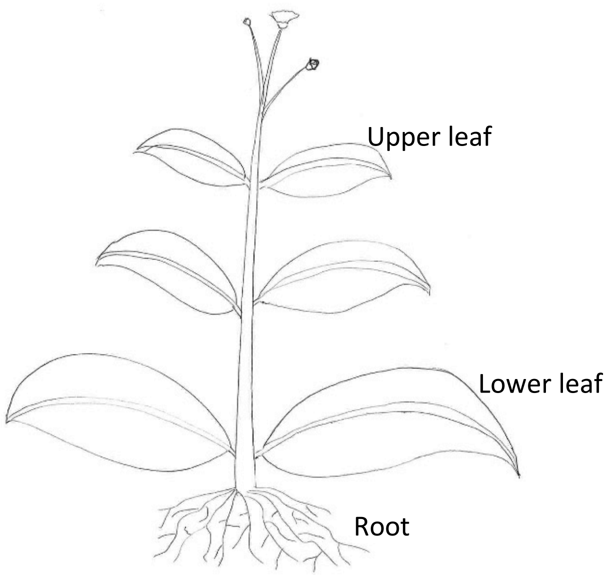

FIELD

4 biological replicates of

- *N. rustica*
- *N. undulata*
- *N. paniculata*
- *N. knightiana*

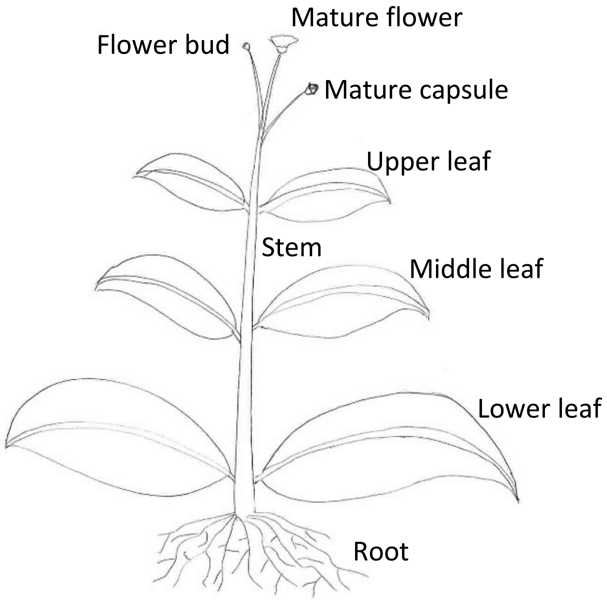

TRANSCRIPTOME

3 biological replicates of

- *N. rustica*
